# Design of Experiments for the Study of High-Power Pulsed Magnetron Sputtering

**DOI:** 10.3390/ma19163515

**Published:** 2026-08-19

**Authors:** Viktor I. Shapovalov, Daniil S. Sharkovskii, Arseny V. Nikolaev

**Affiliations:** Faculty of Electronics, St. Petersburg Electrotechnical University “LETI”, Prof. Popov Str., 5F, St. Petersburg 197022, Russia; sharkovskiy.d@yandex.ru (D.S.S.); nikolaev.a2004@mail.ru (A.V.N.)

**Keywords:** magnetron sputtering, experimental design, full-factorial experiment, fractional-factorial experiment, magnetron target, model, polynomial

## Abstract

The purpose of this study was to conduct an experimental study of gas discharge during high-power pulsed magnetron sputtering of a metal target using a formal experimental design technique (active experiment). The method was applied to sputtering from a predominantly copper target in argon. Discharge voltage was the primary dependent variable; the independent variables were argon pressure, discharge current, pulse duration, and pulse repetition rate. Experiments were carried out on a balanced cylindrical magnetron over a narrow factor range. A first-order polynomial model was found to describe the variable relationships; its adequacy was confirmed by statistical analysis. Using the model, the effect of each factor on pulse power and pulse energy was quantified while holding other factors constant. Pulse fill factor (duty cycle) was introduced as an additional factor. Results from analogous experiments using a titanium target are reported for comparative evaluation.

## 1. Introduction

Magnetron sputtering is a well-established technique for depositing films of diverse materials across a wide range of applications [1]. In a DC magnetron, the density of electrons near the target with mean energies of a few electronvolts does not exceed 10^17^ m^−3^ [2]. Under these conditions, the fraction of ionized atoms in the sputtered flux is only a few percent.

The development of magnetron sputtering techniques led to the development of high-power pulsed magnetron sputtering (HiPIMS). One of the earliest papers on this topic is [3]. High power at the magnetron target was achieved by applying unipolar voltage pulses with a low duty cycle and low repetition rate, producing pulse power at the target roughly two orders of magnitude greater than the average power. The effect exploited by the HiPIMS method was first observed in 1977 [4], when the authors reported a discrepancy between measured and calculated deposition rates for aluminum and copper films; they attributed this to enhanced target sputtering caused by bombardment with ionized sputtered atoms. Because this method has gained broad acceptance, a brief overview is provided.

As shown in numerous subsequent studies [5,6,7,8,9], HiPIMS produces a substantially increased electron density near the target—typically one to two orders of magnitude higher than in DC sputtering. Under these conditions, ionization of sputtered target atoms rises markedly, leading to significant self-sputtering; a fraction of these ions reaches the substrate during film growth. Consequently, HiPIMS enables deposition of high-quality films with strong adhesion, dense microstructure, and enhanced hardness and wear resistance.

Operation in HiPIMS mode can also permit magnetron sputtering in high vacuum without an inert gas. For example, ref. [10] demonstrated that a copper-target magnetron remains stable in self-sputtering mode after the argon supply is turned off. This mode can be potentially useful for depositing metal films.

Detailed studies of the discharge’s current-voltage characteristics revealed two operating modes for a magnetron operating in pulsed mode. For example, at a pressure of 3 mTorr (≈0.4 Pa) and low pulse power density (0.2–0.6 kW cm^−2^), the optical emission spectra of the discharge corresponded to conventional pulsed sputtering. In these spectra, the intensity of argon lines was higher than that of metal lines [11]. At higher powers (2–3 kW cm^−2^), the intensity ratio of metal ion lines to neutral particles in the spectra increased by two orders of magnitude.

**Figure 1:**
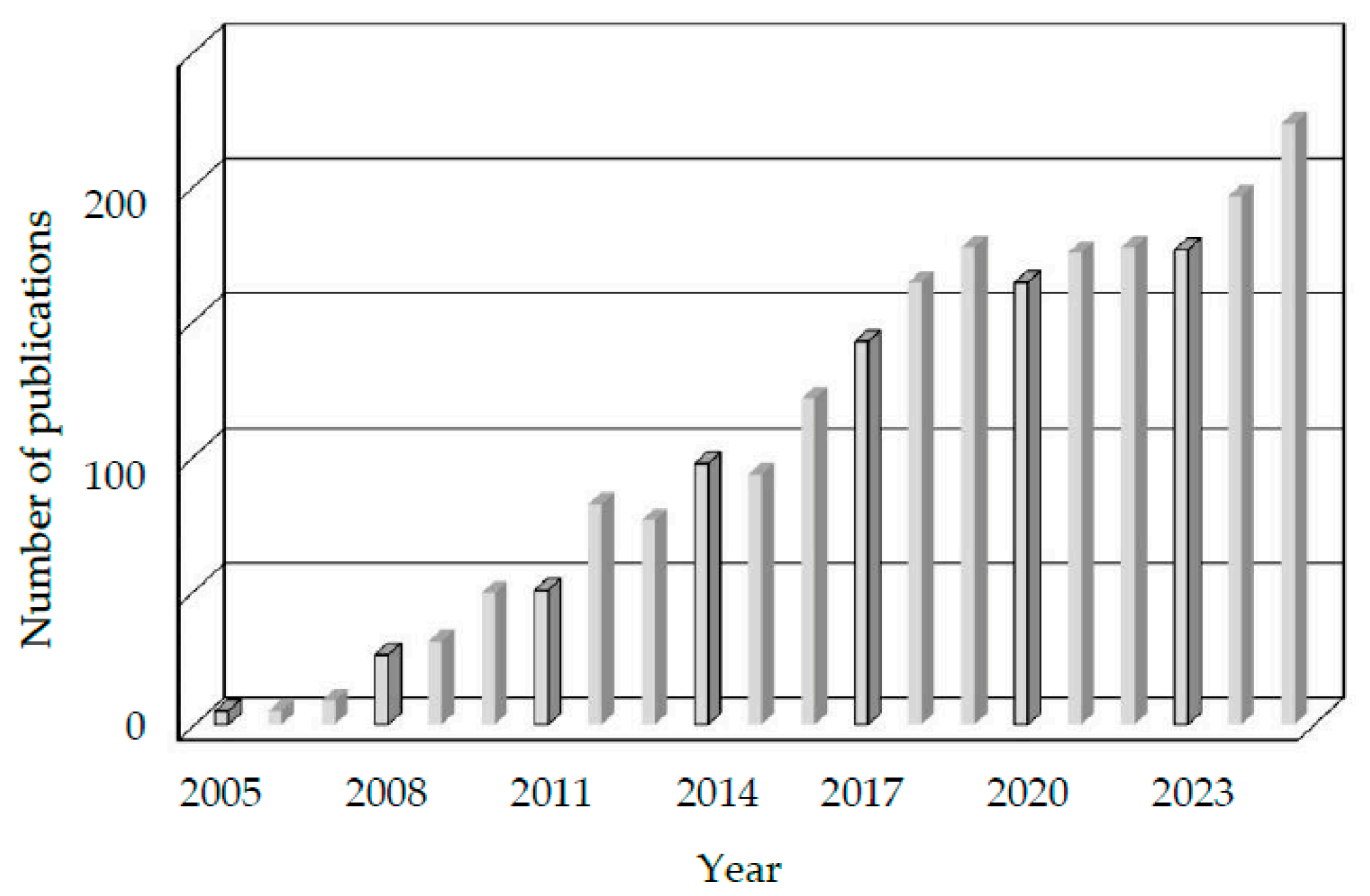
Publications on high-power pulsed magnetron sputtering (keyword “HiPIMS”) from 2005 to 2025, based on Scopus.

HiPIMS has been applied beyond single-element film deposition; several studies have demonstrated its suitability for producing equiatomic metal-alloy films [12,13,14,15,16,17]. Periodic review articles [18,19,20,21] compile extensive bibliographies covering HiPIMS under various conditions, and Figure 1 illustrates the method’s rising interest.

For synthesis of simple compound films and their solid solutions (oxides, nitrides, etc.) by HiPIMS [22,23,24,25,26,27,28,29,30], nonlinear phenomena characteristic of DC reactive sputtering are likewise observed; this mode is commonly denoted R-HiPIMS. Unlike metal sputtering in argon, R-HiPIMS exhibits additional target sputtering by reactive-gas ions, which becomes more pronounced as the discharge current increases in the reactive mode [24].

HiPIMS is a multifactorial process; therefore, the initial goal of any study of this method is to examine the influence of key factors on the final result. This result can be expressed as, for example, discharge voltage, pulse power or energy, film growth rate, and film properties. Among the factors, authors often limit their interest to only the pulse duration τ [31,32]. In other cases, they include the pulse repetition rate *f* or the fill factor *S* = 1/τ*f* (duty cycle *D* = τ*f*) [9,30]. These factors are the most significant, but for a more detailed study of the sputtering process in an argon environment, this list is supplemented by discharge pressure and current [5,6]. In more complex cases, the set of factors includes temperature and substrate bias potential [29]. This creates a six-dimensional factor space, into which the reactive gas flow rate is added when using the R-HiPIMS method.

A common tactic for investigating multifactorial processes like HiPIMS is to run multiple experimental series in which a single factor is varied across several levels while the others are held constant [5]. This approach is practical for a small number of factors but becomes impractical as dimensionality grows: for example, three factors with four levels each require 4^3^ experiments, and the effort is infeasible for seven factors. In such cases, formal experimental design methods are used, as is done for bipolar pulse-reactive magnetron sputtering [33] with three independent variables and in other cases [34,35].

This approach allows us, firstly, to reduce the number of experiments. Secondly, it is used to establish an analytical description of the process under study within a multidimensional experimental domain. This makes it possible to predict the values of the dependent variable at any point in a given area.

In this paper, elements of the theory of experimental planning were used to study gas discharge during high-power pulsed magnetron sputtering of a metal target. The discharge voltage was assumed to be the main dependent variable. The independent variables were argon pressure, discharge current, pulse repetition duration and frequency. The authors are not aware of works in which this multidimensional dependence was obtained in an analytical form.

**Figure 2:**
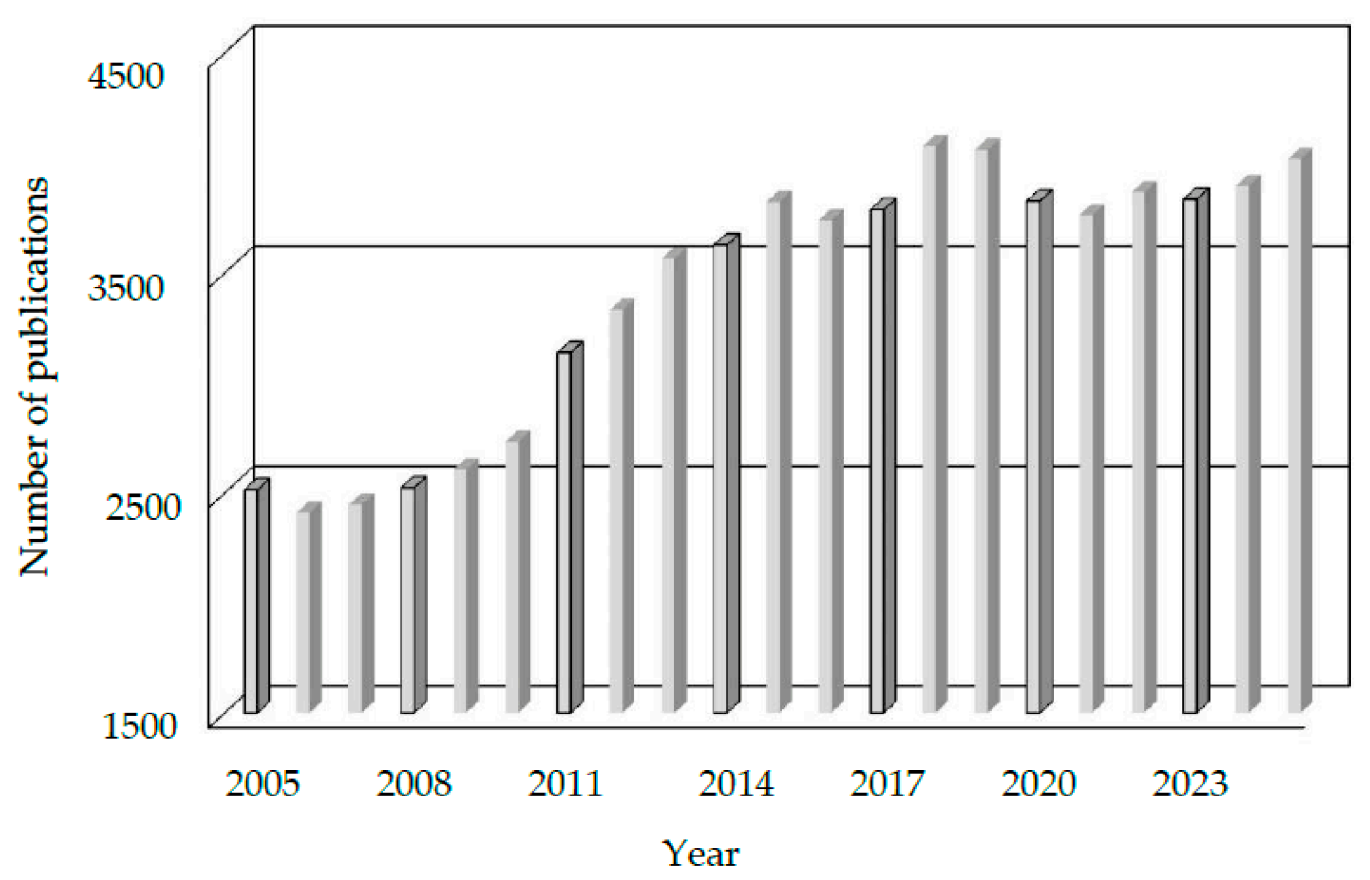
Publications on copper films (keywords “film and Cu or copper”) from 2005 to 2025 based on Scopus.

The studies were performed for a copper target. Copper films and their simple compounds have long attracted interest because of their functional properties. Over the past decade, publications on these films have stabilized at a level exceeding 3500 articles (see Figure 2). Comprehensive studies are reviewed in [31,32,36,37,38,39,40,41].

Pulse fill factor (duty cycle) was introduced as an additional factor. Results from analogous experiments using a titanium target are reported for comparative evaluation.

## 2. Basic Concepts of Experimental Design

Possible strategies for a physical experiment will be considered. To do so, we will refer to the object of interest as the system. The experimenter is assumed to control the system by varying a set of independent input variables (factors), while one or more output variables depend on those factors. For well-structured systems, the experimental results can be expressed as unambiguous functional relationships between inputs and outputs that effectively represent physical laws. Gas discharges and devices based on them do not belong to this class: a discharge comprises many elementary, interdependent processes that cannot be uniquely identified and described mathematically. Consequently, the experimenter must resort to modeling. Two model types are possible.

A physical model is obtained by simplifying the system: the principal elementary processes are identified and described using established laws, yielding a system of equations whose solution reveals characteristic relationships among variables. The model’s adequacy is then tested against experimental data using standard statistical methods.

Another approach is being developed within cybernetics, where a complex system is represented as a “black box” with input and output variables. In this type of model, the relationships between variables are typically postulated as polynomials. Such models are called regression equations. They are based on the ability to represent an arbitrarily complex function in the neighborhood of a point in the form of a series, such as a Taylor series. For example, given three independent variables *X*_1_, *X*_2_, *X*_3_ and one dependent variable *y*, a first-order polynomial can be used:

(1) $$y=\beta_{0}+\beta_{1}X_{1}+\beta_{2}X_{2}+\beta_{3}X_{3}.$$

where β*_i_ i* = 0, 1, 2, 3 are the unknown model coefficients that should be estimated based on the experimental results.

The adopted model should be regarded as a hypothesis subject to experimental verification. Designing the verification experiment is the central problem when applying a cybernetic model. To address this, a mathematical theory of experimentation was developed within mathematical statistics [42,43,44].

A key concept of this theory is the optimal use of the independent-variable space, which leads to multifactor experiments in which the unknown model coefficients are estimated from the entire set of runs, thereby reducing estimation error. During the experiment, all independent variables are varied simultaneously when moving from one run to the next, introducing controlled randomness; this procedure—randomization—means assigning a random order to the runs in the chosen design.

We will limit the algorithm for constructing a cybernetic model to several steps:

(1) selection of the model;
(2) selection of the experimental design;
(3) execution of the experiment;
(4) estimation of the model coefficients;
(5) assessment of model adequacy.

Typically, to design an experiment and calculate model coefficients, dimensional independent variables are normalized. The normalization procedure is described in more detail below. If model (1) is adopted, then in the normalized coordinate system it takes the form

(2) $$y=b_{0}+b_{1}x_{1}+b_{2}x_{2}+b_{3}x_{3}.$$

where *b_i_* and *x_i_* (*i* = 0, 1, 2, 3) are the estimates of the model coefficients and normalized independent variables, respectively.

For model (2), each factor in the experimental design can be varied at two levels. In the new dimensionless coordinate system, the highest value of each physical variable corresponds to “+1,” and the lowest to “−1.” Clearly, a design containing all possible combinations of values, for example, for *k* factors, must consist of 2*^k^* different experiments. This is called a full-factorial design (FFD) [45]. As the number of factors increases, FFDs typically become redundant. In these designs, the number of experiments exceeds the number of unknown coefficients in the model. The experimental design method allows for the possibility of reducing the number of experiments by using a fractional-factorial design (FrFD) [45]. The experimental design is defined by a rectangular matrix. For model (2), it will have four columns. The number of rows in this matrix is determined by the number of different experiments planned. For model (2), the FFD must contain 2^3^ = 8 experiments.

The experimental result contains a series of values of the dependent variable. The least squares method is used to calculate the model coefficients. This leads to a system of equations, the solution of which in the matrix has the following form [45]:

(3) $$b={\left(X^{T}X\right)}^{-1}X^{T}Y.$$

where **b** is a column vector of model coefficient estimates; **X** is the design matrix; T is the transpose sign; **Y** is a column vector of the measurement results of the dependent variable *y*. If the model contains *k* variables and the experiment consists of *N* different runs, then the matrix **X** will have size *N* × (*k* + 1), and the vector **Y** will have size *N* × 1. The first column of the matrix **X** corresponds to the dummy variable *x*_0_ ≡ 1. It is added to **X** to estimate the intercept b_0_ in model (2). The product **X**^T^**X** is called the information matrix.

The 2*^k^*-type FFD has the orthogonality property, which means that

(4) $$\sum_{i=1}^{i=N}x_{ij}x_{iu},\;j,\;u=0,\;1,\;\ldots ,\;k,\;j\neq \;u.$$

For such a plan, the information matrix **X**^T^**X** is not only diagonal, but also expressed through the identity matrix **I**:

**X**^T^**X** = *N***I**. (5)

The orthogonality of the design significantly simplifies the calculation of the model coefficient estimates. Matrix expression (3) breaks it down into a series of formulas:

(6) $$b_{0}=\frac{1}{N}\sum_{i=1}^{i=N}y_{i},\;b_{j}=\frac{1}{N}\sum_{i=1}^{i=N}x_{ij}y_{i},\;j=1,\;2,\ldots ,\;k.$$

## 3. Experiment

The experimental object was a cylindrical balanced magnetron with a diameter of 130 mm, equipped with a 6 mm thick, 99.9% pure copper target. The study was conducted in a vacuum chamber with a volume of 7.8 × 10^−2^ m^3^ at a residual pressure of no more than 10^−3^ Pa. High vacuum in the chamber was maintained by a diffusion pump and a rotary vane pump with pumping speeds of 300 and 0.005 m^3^/s, respectively. The magnetron was powered by an APEL-M-5HIPMS-1K pulsed power supply (Applied Electronics, Tomsk, Russia), capable of a maximum power of up to 5 kW at a voltage of up to 1000 V, and a maximum output current in a pulse of up to 1 kA with a current stabilization accuracy of up to ±3%. The source generates pulses with a duration of 5–250 mks with a frequency range of 10 Hz–15 kHz and a step interval of 10–100 Hz.

The shape of the current pulses generated by this source with rectangular voltage pulses (similar to the case in [27]) varies from triangular to trapezoidal, depending on their duration. The average current value is used as the independent variable in the work. At the same time, the average amplitude value is assumed for the voltage. The microcontroller in the power supply fixes the voltage based on the storage capacity of the pulse generator. Its value is displayed on a digital indicator. The charge current is simultaneously measured and displayed on the indicator (current sensor LAH25-NP, LEM, Milwaukee, WI, USA). It is actually the average current, which we have accepted as one of the independent variables.

The experiment was conducted in three stages. Preliminary experiments in the first stage aimed to identify the features of the current-voltage characteristic (I–V characteristics) of the discharge. The I–V characteristic was varied by varying the discharge current in the range of 0.5–2.0 A in 0.25 A increments, at τ = 50, 60, 80, and 100 μs and constant values of *p* = 0.7 Pa and *f* = 1500 Hz.

The results made it possible to postulate the form of the mathematical model. In the second stage, experiments were conducted according to the design corresponding to the chosen model, using the factor ranges listed in Table 1. In the third stage, five repeated experiments were performed with constant values of all factors: *p* = 0.7 Pa, *I* = 1.2 A, τ = 90 μs, and *f* = 1500 Hz. Based on the results of these measurements, the variance of the pure (random) experimental error was estimated.

To further demonstrate the capabilities of the proposed method, an active experiment was conducted to study the sputtering process of a titanium target. This process differed from the one described above only in that a 6 mm thick, 99.9% pure titanium target was mounted on the magnetron. The experimental methodology, including its design, shown in Table 1, remained unchanged.

## 4. Model of High-Power Pulsed Magnetron Sputtering

The objective of the study was to determine the influence of four independent variables on magnetron discharge: argon pressure *p*, average discharge current *I*, duration τ, and pulse repetition rate *f*. The discharge voltage was used as the dependent variable.

**Figure 3:**
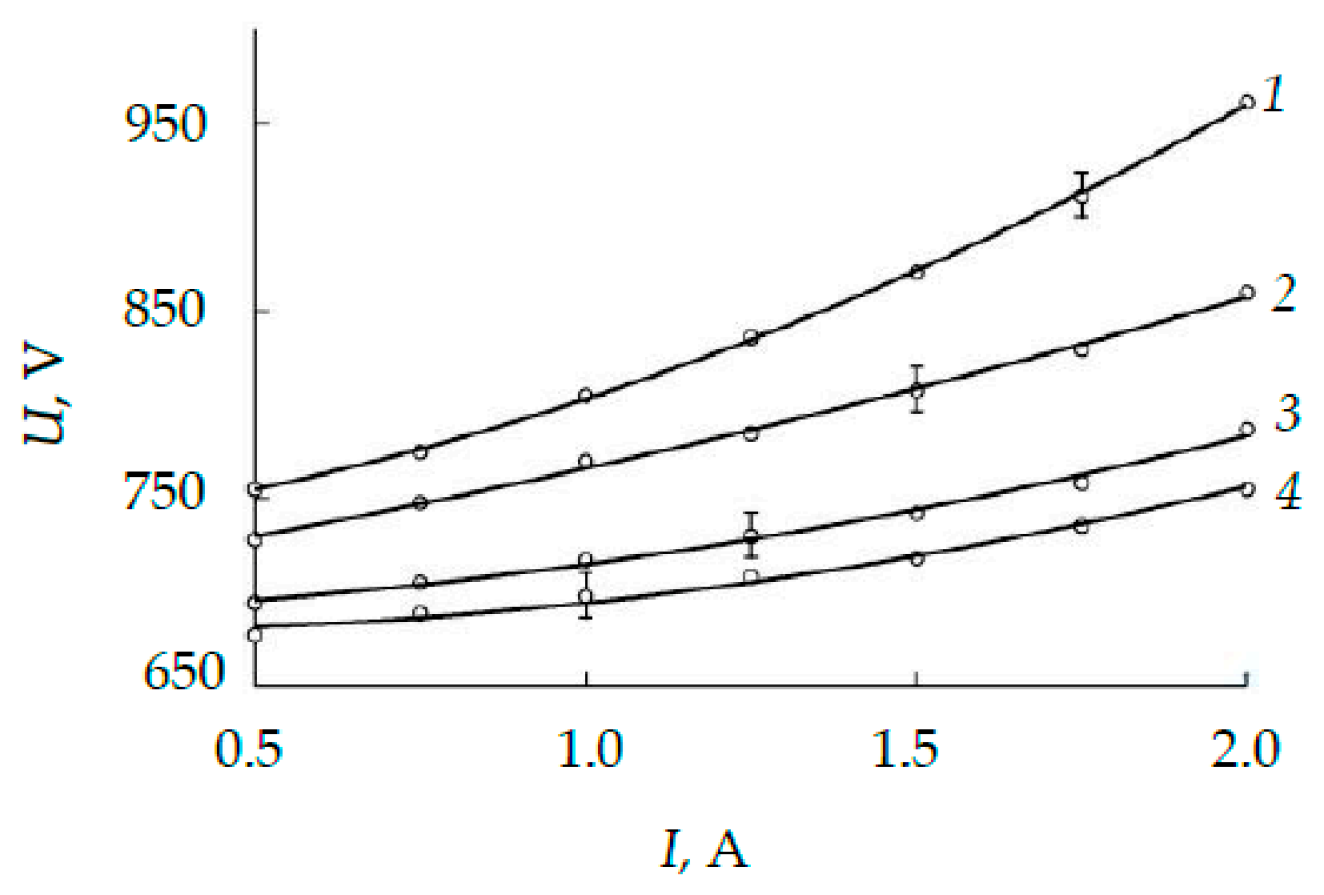
I–V characteristics of the magnetron at *p* = 0.7 Pa, *f* = 1000 Hz and τ (in μs): *1*—50; *2*—60; *3*—75; *4*—100 (dots—experiment, solid lines—trend).

**Figure 4:**
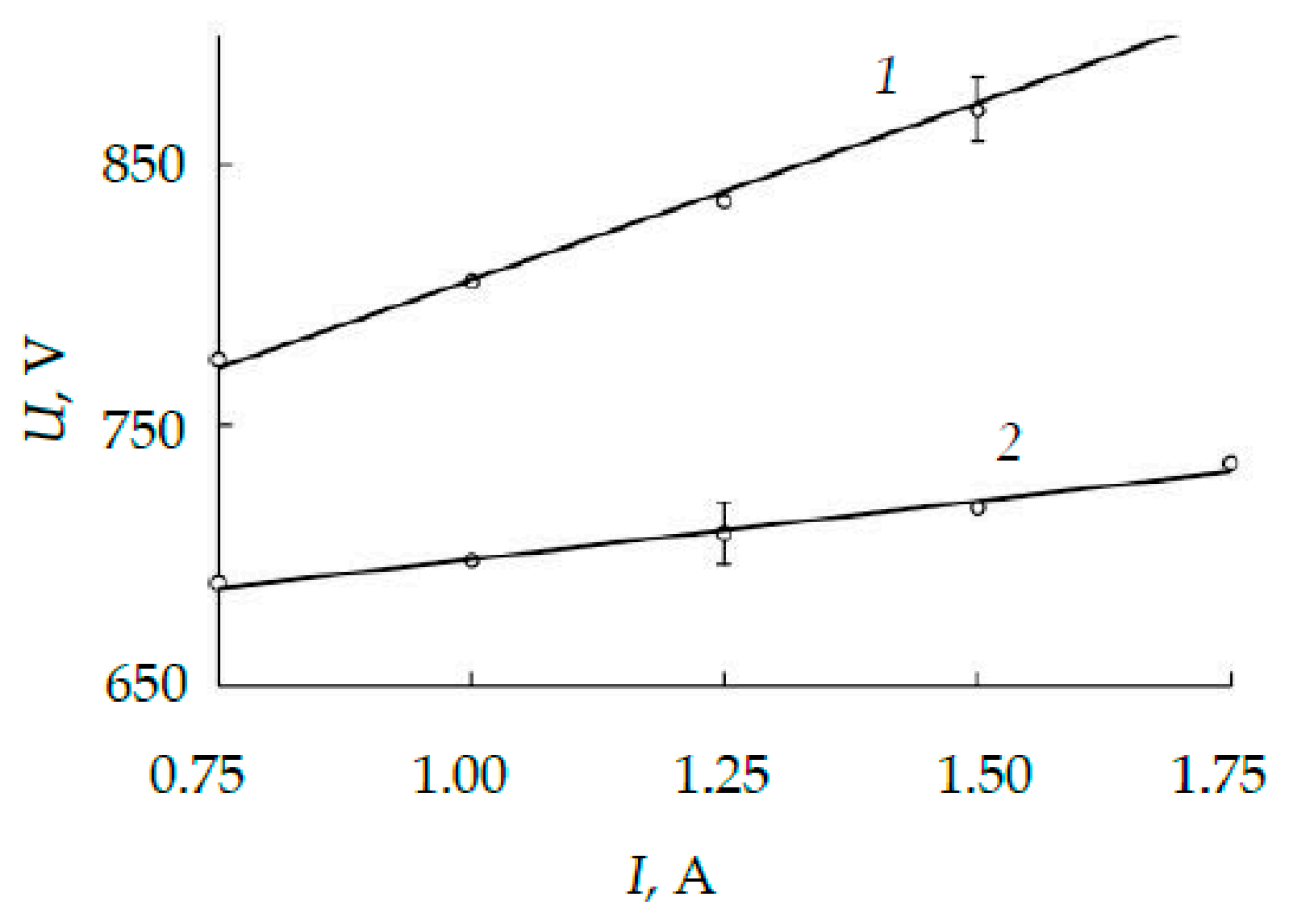
I–V characteristics of the magnetron at *p* = 0.7 Pa, *f* = 1000 Hz and τ (in μs): *1*—50; *2*—100 (dots—experiment, solid lines—trend).

Preliminary experiments served as the basis for the design of the study. Figure 3 partially reflects their results. The I–V characteristics in Figure 3 suggest that a first-order polynomial can be used to approximate the I–V characteristics in the range from 0.75 to 1.75 A. This is illustrated in Figure 4, where the reliability of this approximation is 0.996 for τ = 50 μs (line 1), and 0.982 for τ = 100 μs (line 2).

**Figure 5:**
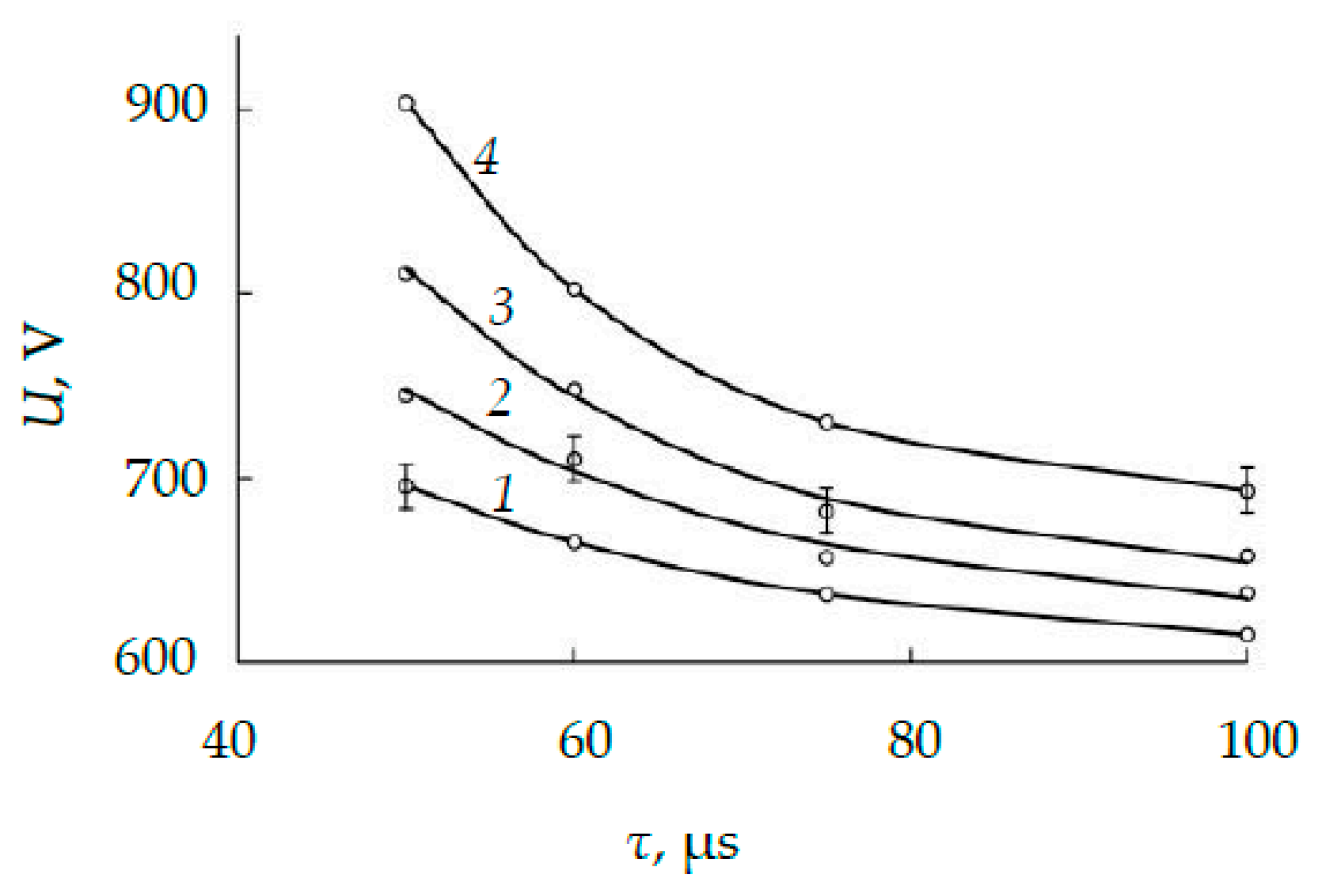
Dependences of the discharge voltage on the pulse duration at *p* = 0.7 Pa, *f* = 1000 Hz and *I* (in A): *1*—0.5; *2*—1.0; *3*—1.5; *4*—2.0 (dots—experiment, solid lines—trend).

The results shown in Figure 5 demonstrate the validity of this approximation for pulse durations greater than 70 μs. This provides grounds for adopting the dependence *U* = *F*(*p*, *I*, τ, *f*) as a mathematical model in the form

(7) $$U=F\left(p,\;I,\;\tau ,\;f\right)=\beta_{0}+\beta_{1}p+\beta_{2}I+\beta_{3}\tau +\beta_{4}f,$$

where the value β_0_ can be interpreted as the coefficient of a variable *X*_0_ ≡ 1, which is usually called the dummy.

To determine the unknown coefficients β*_j_ j* = 0, 1, 2, 3, 4 in model (7), the experiment mentioned in Section 2 was designed and carried out. The region of the space of independent variables in which function (7) is defined for this case is described using Table 1. To develop the design, the physical variables *p*, *I*, τ and *f* were transformed into dimensionless *x_j_, j* = 1, 2, 3, 4:

(8) $$x_{j}=\frac{X_{j}-X_{j0}}{\Delta_{j}},$$

where *X_j_*_0_ and Δ*_j_* are the central point of the region (center of the plan) and the variation interval of the *j*-th variable

(9) $$X_{j0}=\frac{X_{j\max}+X_{j\min}}{2},\;\Delta_{j}=\frac{X_{j\max}-X_{j\min}}{2}.$$

Transformation of coordinates using (8) and (9) led to a mathematical model:

(10) $$U=b_{0}+b_{1}x_{1}+b_{2}x_{2}+b_{3}x_{3}+b_{4}x_{4}.$$

The FFD for model (10) contains 2^4^ = 16 experiments. It is redundant, as it is designed to calculate estimates for only five coefficients of model (10). Therefore, a semi-replica of the FFD, containing 2^4−1^ = 8 experiments, was adopted for the experiment. This FrFD is specified in Table 2. We will further discuss some details of its development.

The core of the FrFD in Table 2 is the FFD of type 2^3^. It is listed in columns *x*_1_, *x*_2_ and *x*_3_. Each row of these three columns defines the conditions for performing a single experiment. The design is obtained by exhaustively searching through combinations of values of three variables and defines a hypercube-shaped domain in three-dimensional space.

Conducting an experiment using a type 2^3^ design allows us to construct a model of type (2). However, we have not written it down completely. It lacks components that reflect possible interactions between variables. In full form, model (10) in normalized variables is given by

(11) $$U=b_{0}+b_{1}x_{1}+b_{2}x_{2}+b_{3}x_{3}+b_{12}x_{1}x_{2}+b_{13}x_{1}x_{3}+b_{23}x_{2}x_{3}+b_{123}x_{1}x_{2}x_{3}.$$

Expression (11) adds three components reflecting possible pairwise interactions between variables with coefficients *b*_12_, *b*_13_, and *b*_23_ and one triple interaction with coefficient *b*_123_. Our simplification of model (11) is based on the assumption that the interactions between the adopted independent variables are insignificant. Indeed, for example, the argon pressure in the chamber cannot be related to the pulse repetition rate. This also applies to other pairs of parameters.

The simplified model (11) allowed *x*_4_ to be varied in the 2^4−1^ FrFD so as to emulate any interaction term in the 2^3^ FFD. In Table 2, this substitution is adopted as *x*_4_ = *x*_1_*x*_3_.

The first column of the plan in Table 2 corresponds to the dummy variable *x*_0_ and is only needed for calculations using formula (3). The last column contains the discharge voltage measurement results for each experiment.

After completing the experiment according to the given plan, the coefficient estimates for model (10) were calculated using expression (3). The initial data were the planning matrix **X** and the column vector of measurement results **Y**. Since the experiment measured a real physical variable, we replace the vector **Y** in (3) with U*:

(12) $$X=\left(\begin{array}{ccccc} 1 & -1 & -1 & -1 & 1\\ 1 & -1 & -1 & 1 & -1\\ 1 & -1 & 1 & -1 & 1\\ 1 & -1 & 1 & 1 & -1\\ 1 & 1 & -1 & -1 & -1\\ 1 & 1 & -1 & 1 & 1\\ 1 & 1 & 1 & -1 & -1\\ 1 & 1 & 1 & 1 & 1 \end{array}\right),\;U^{*}=\left(\begin{array}{c} 676\\ 682\\ 717\\ 717\\ 668\\ 610\\ 716\\ 654 \end{array}\right).$$

The components of the vector U* in (12) are in units of volts.

It is easy to verify that the matrix **X** we used is orthogonal. This is confirmed by product (5):

(13) $$\left(X^{T}X\right)=\left(\begin{array}{ccccc} 8 & 0 & 0 & 0 & 0\\ 0 & 8 & 0 & 0 & 0\\ 0 & 0 & 8 & 0 & 0\\ 0 & 0 & 0 & 8 & 0\\ 0 & 0 & 0 & 0 & 8 \end{array}\right)=8I.$$

The result in Formula (13) allows us to use the simple Formula (6) for calculations. Thus, with initial data in Formula (12), we obtained estimates of the model coefficients. Model (10) then takes the form

*U* = 680 − 18.0*x*_1_ + 21.0*x*_2_ − 14.3*x*_3_ − 15.8*x*_4_. (14)

Obviously, the sign before each term in (14) uniquely defines the influence of a given factor on the value of *U*: a minus sign (for *x*_1_, *x*_3_ and *x*_4_) indicates a decrease in *U* with an increase in a given factor, while a plus sign (for *x*_2_) indicates an increase. Note that the variables in model (14) are dimensionless. This means that all coefficients must be expressed in volts. If necessary, model (14) can be rewritten using real physical variables. To do this, expression (8) is substituted into (14) and the necessary transformations are performed. In this case, model (7) will be obtained, and its coefficients will acquire different dimensions. The exception is the coefficient β_0_ for the dummy variable *X*_0_ ≡ 1, expressed in volts.

The quality of model (14) was determined by its deviation from the points of the actual experiment [45]:

(15) $$\sigma_{\mathrm{ad}}^{2}=\frac{1}{N-(k+1)}\sum_{i=1}^{i=N}{\left(U_{i}-U_{i}^{*}\right)}^{2},$$

where $U_{i}$ is the value calculated using model (14); $U_{i}^{*}$ is the measured value from the vector U*. The quantity $\sigma_{\mathrm{ad}}^{2}$ in (15) is called the adequacy variance. Calculations using Formula (15) are summarized in Table 3.

Next, we need to determine the following: is it good or bad if $\sigma_{\mathrm{ad}}^{2}\approx 15$? In other words, we need to check the model’s conformity to the experiment, or its adequacy. This check consists of establishing the nature of the quantity $\sigma_{\mathrm{ad}}^{2}$. It could appear due either to random experimental error or to model inadequacy.

It follows that the experiment cannot be limited to the design specified in Table 2. Analyzing the adequacy of the model requires knowledge of the random error of the experiment. Assuming that the measured dependent variable is subject to a random error with zero mathematical expectation, its properties are specified by variance, which we denote as $\sigma_{U}^{2}$. To estimate it, it is sufficient to perform a series of trials at a single point in the experimental domain. This is correct if there is confidence that the random error is constant at all points in the experimental domain. We have no reason to assume otherwise. Therefore, we performed additional experiments at the central point of the design (with all dimensionless variables equal to zero). Five repeated experiments at this point allowed us to establish that $\sigma_{U}^{2}\approx 72$.

Next, we need to solve a very simple problem. The adequacy of the model is tested using Fisher’s exact test [46], calculating the ratio $F=\sigma_{\mathrm{ad}}^{2}/\sigma_{U}^{2}$. In our case, since the variance of the adequacy is less than the variance of the random error, the value F<1. In this case, the adequacy testing procedure can be concluded by concluding that the model is acceptable. In other words, the adequacy of the model does not contradict the experimental data. We emphasize that this conclusion is obtained under the assumption that random error is constant in the experimental domain.

The result obtained for the model that describes the discharge voltage will also be valid for other dependent variables related to the discharge voltage. These include, for example, the power P=UI/τf and energy E=Pτ in the pulse. This means that model (14) can also be used to predict the values of the specified quantities. However, this is only true for the region of the factorial experiment, which is specified in Table 1. The prediction error when extrapolating beyond this region is unknown.

**Figure 6:**
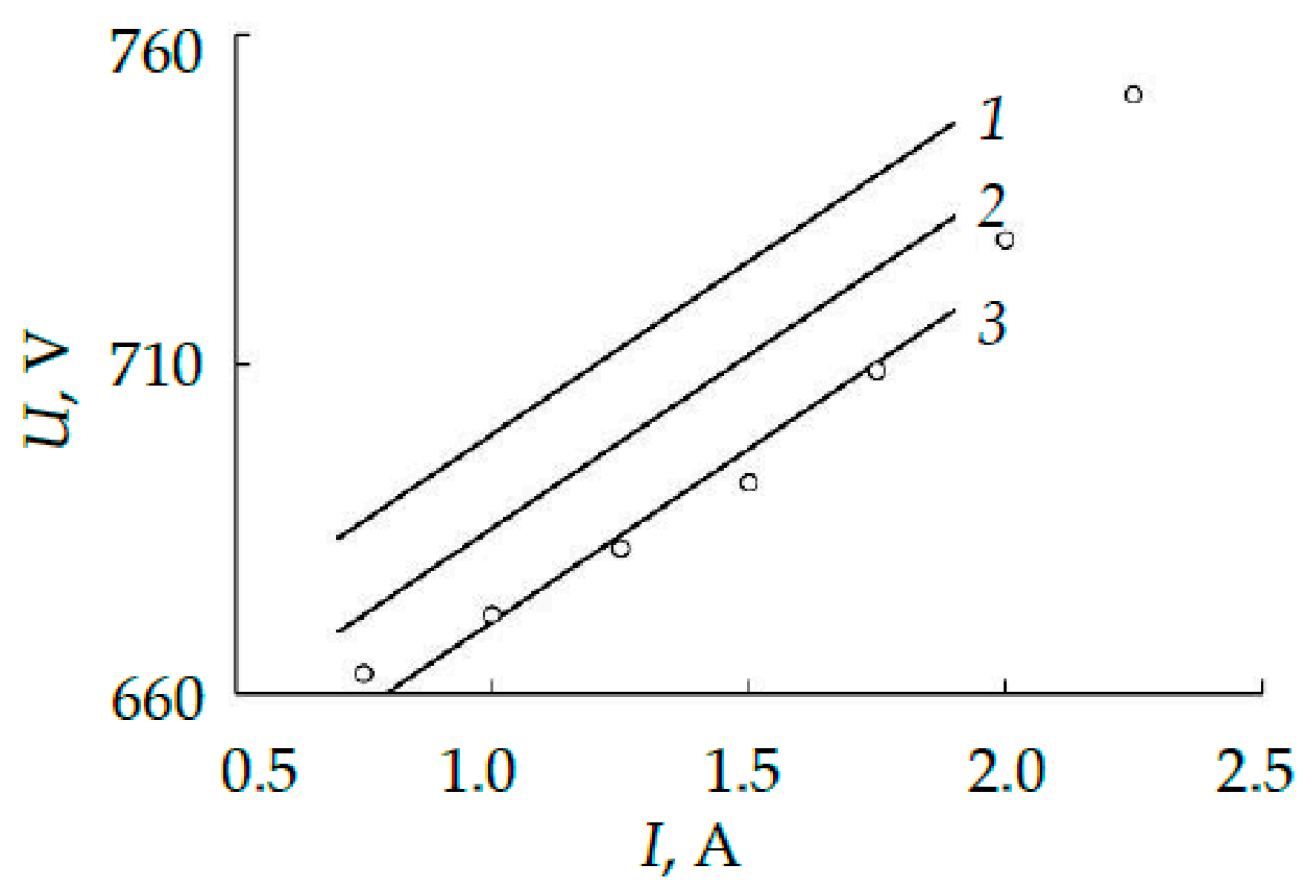
I–V characteristics of the magnetron at *p* = 0.7 Pa, *f* = 1000 Hz. Dots represent the experiment at τ = 100 μs. Solid lines represent the model at τ (in μs): *1*—80; *2*—90; *3*—100.

In Figure 6, the solid lines for the region of the factorial experiment (0.8–1.6) A plot the model’s I–V characteristics. The dots in Figure 6 show the results of a separate experiment carried out in the first series in a wider range, with the values of independent variables indicated in the caption.

The difference between the experimental and model (line 3) dependences was determined by the standard deviation between them:

(16) $$\delta_{U}^{2}=\frac{1}{5}\sum_{i=1}^{i=5}{\left(U_{i}-U_{i}^{*}\right)}^{2},$$

where, unlike in (15), $U_{i}\;i=1,\;2,\ldots ,\;5$ and $U_{i}^{*}i=1,\;2,\ldots ,\;5$ are the values calculated using model (14) and measured in the first series, respectively, at the current values established in the first series and falling within the factorial experiment range. The value of (16), calculated in the range of (0.8–1.6) A, did not exceed 15 V^2^, resulting in a relative error of no more than ±3% with a confidence level of 0.997. This result can be considered acceptable.

**Figure 7:**
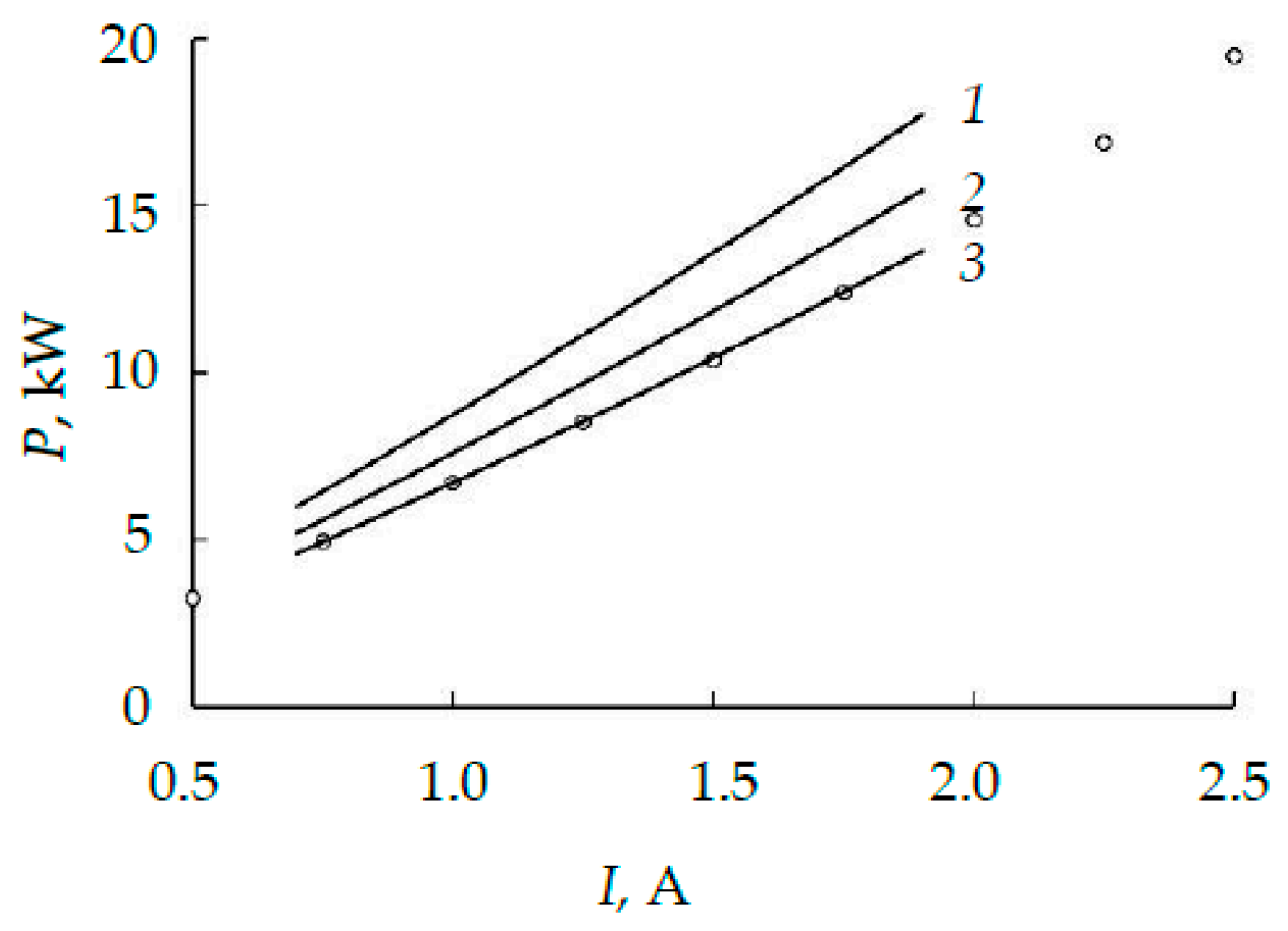
Pulse power versus discharge current at *p* = 0.7 Pa and *f* = 1000 Hz. Dots represent the experiment at τ = 100 μs. Solid lines represent the model at τ (in μs): *1*—80; *2*—90; *3*—100.

Figure 7 illustrates the application of model (14) to predict the effect of discharge current on pulse power in the form of dependences $P=U\left(I\right)I/\tau f=f{\left(I\right)}_{p,\;\tau ,\;f=\mathrm{const}}$. The dots in Figure 7, as in Figure 6, represent the results obtained in the first series of the experiment. The difference between the experimental and model (line 3) dependences, calculated by analogy with (16), is approximately ± 1.5%.

**Figure 8:**
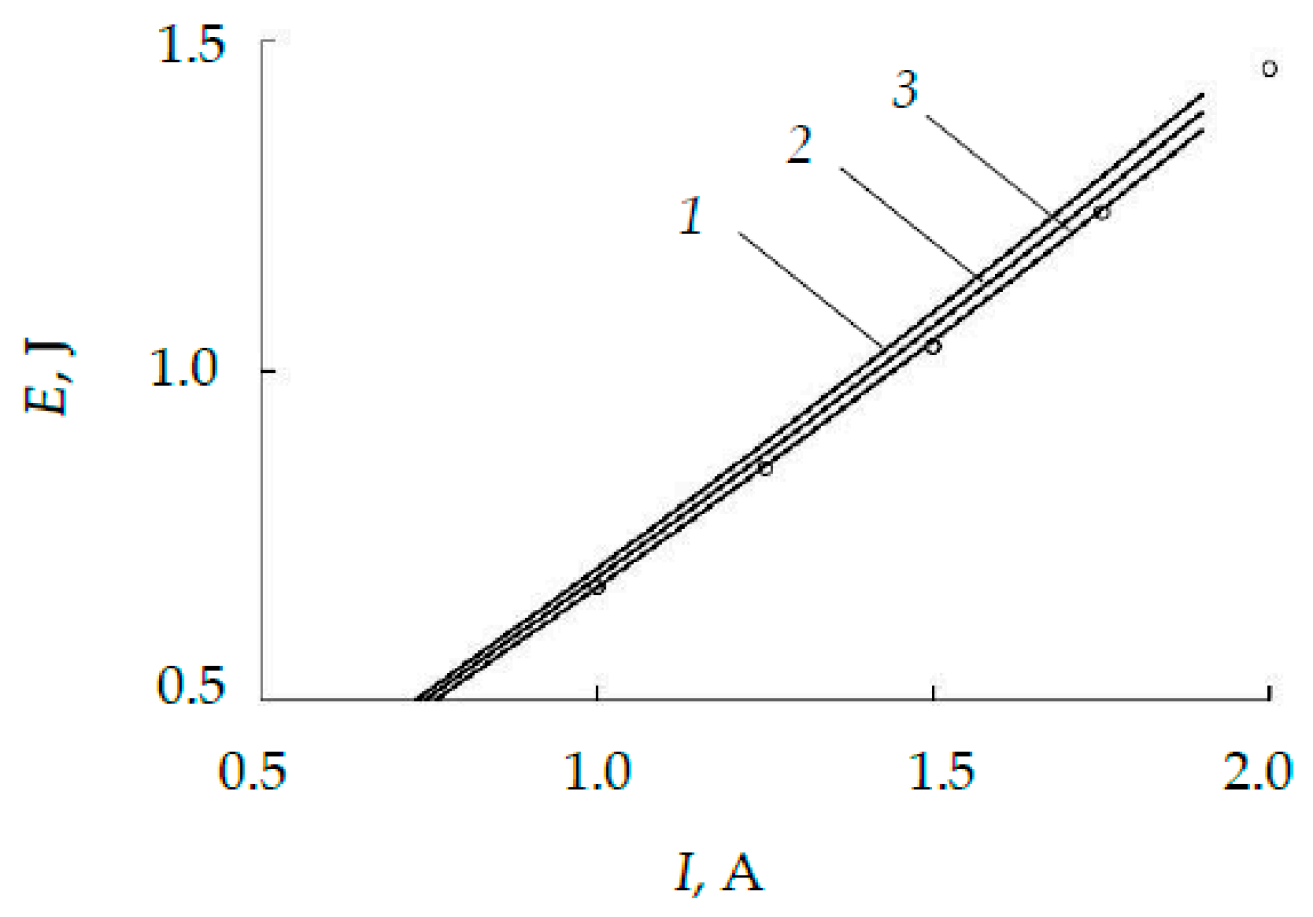
Pulse energy as a function of discharge current at *p* = 0.7 Pa and *f* = 1000 Hz. Dots represent the experiment at τ = 100 μs. Solid lines represent the model at τ (in μs): *1*—80; *2*—90; *3*—100.

Figure 8 shows the influence of discharge current on pulse energy, studied using model (14). The experimental results are shown in Figure 8 as dots. The model dependences are given in the form $E=f{\left(I\right)}_{p,\;\tau ,\;f=\mathrm{const}}$. The difference between the experimental and model (line 3) dependences, calculated by analogy with (16), does not exceed ±1.0%. Figure 6, Figure 7 and Figure 8 demonstrate excellent interpolation of the studied dependences with discharge current, performed in the context of a factorial experiment using model (14).

This model makes it possible to study the influence of other factors characteristic of high-power magnetron sputtering on dependent variables. These include, for example, the dependences of the discharge voltage $U=f{\left(\tau \right)}_{p,\;I,\;f=\mathrm{const}}$ or pulse power on pulse duration $P=f{\left(\tau \right)}_{p,\;I,\;f=\mathrm{const}}$.

**Figure 9:**
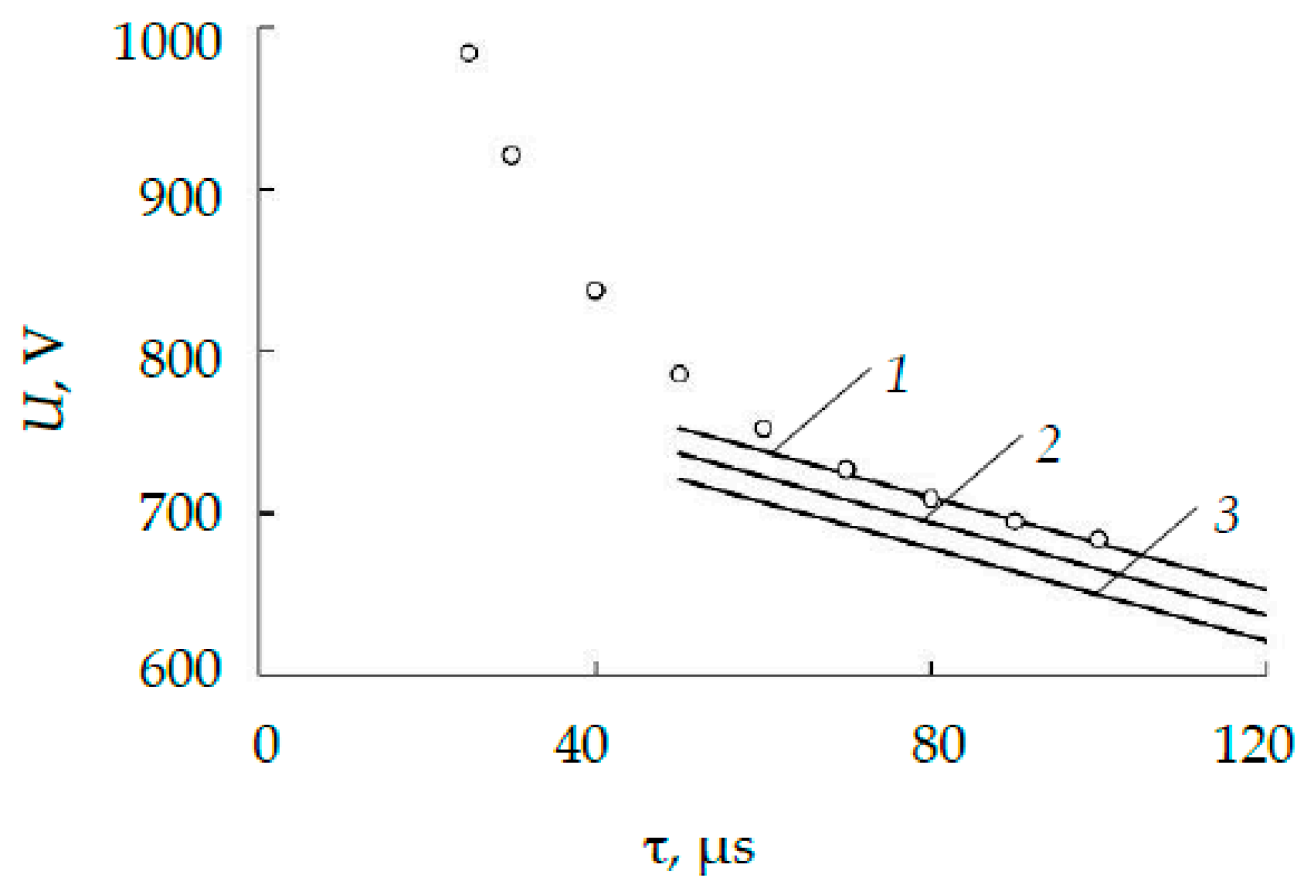
Dependences of the discharge voltage on the pulse duration during sputtering of a copper target at *p* = 0.7 Pa, *I* = 1.2 A. Dots—experiment at *f* = 1000 Hz. Solid lines—model at *f* (in Hz): *1*—1000; *2*—1500; *3*—2000.

For example, in Figure 9, the solid lines show the model dependences reflecting the influence of pulse duration on discharge voltage at constant values of argon pressure, discharge current, and pulse repetition rate. The dots in Figure 9 show the results of a separate experiment. Without using expression (14), it is clear from Figure 9 that the model at a pulse repetition rate of 1000 Hz is close to the experimental results. However, this closeness is observed only in the experimental region (at 80 ≤ τ ≤ 100 μs). At shorter pulse durations, the experimental dependence $U=f{\left(\tau \right)}_{p,\;I,\;f=\mathrm{const}}$ exhibits significant nonlinearity.

**Figure 10:**
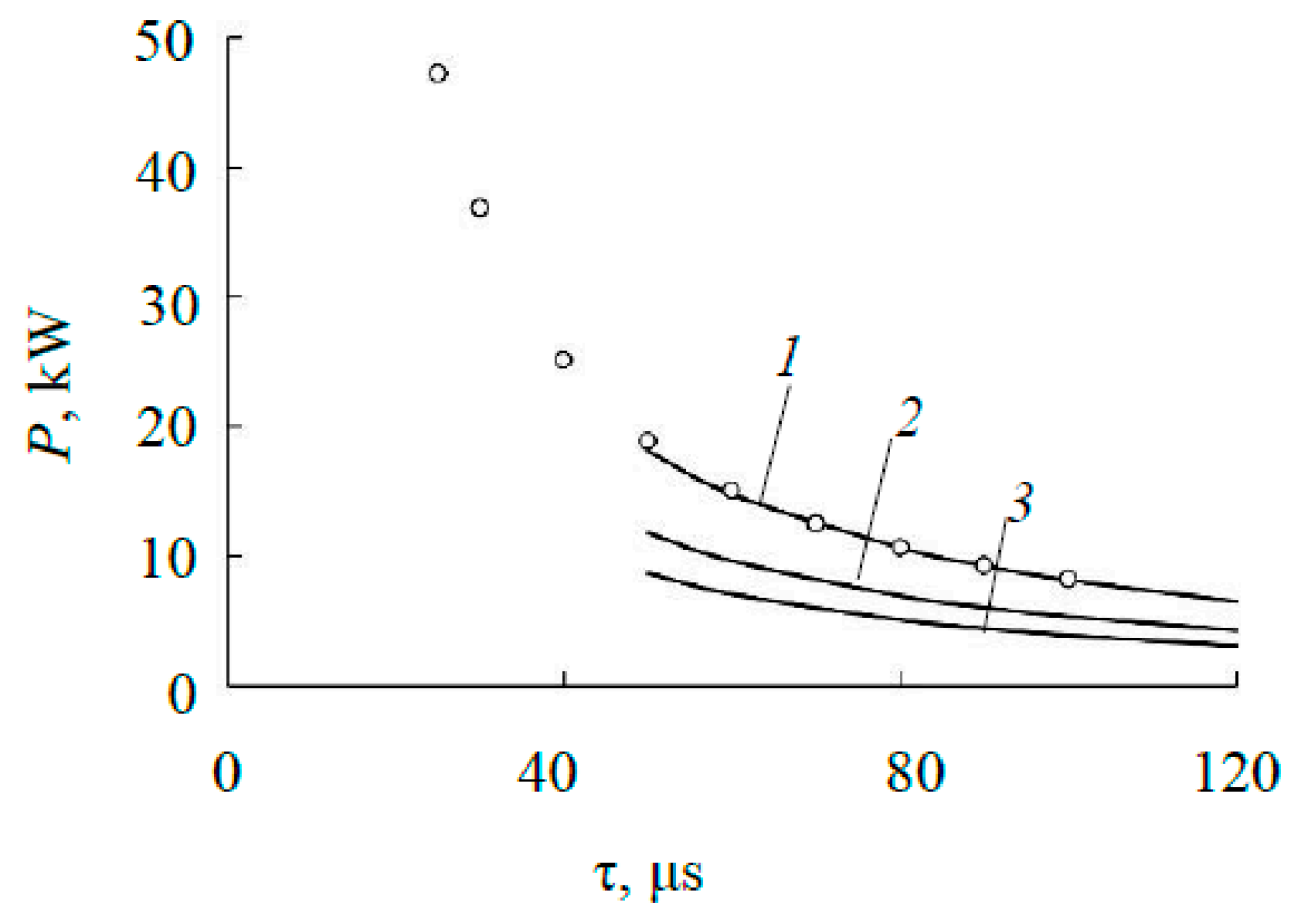
Pulse power versus pulse duration during sputtering of a copper target at *p* = 0.7 Pa and *I* = 1.2 A. Dots represent the experiment at *f* = 1000 Hz. Solid lines represent the model at *f* (in Hz): *1*—1000; *2*—1500; *3*—2000.

A similar result is characteristic of the dependences $P=f{\left(\tau \right)}_{p,\;I,\;f=\mathrm{const}}$ shown in Figure 10. Here, model (14) also deviates significantly from the experiment at τ < 80 μs.

**Figure 11:**
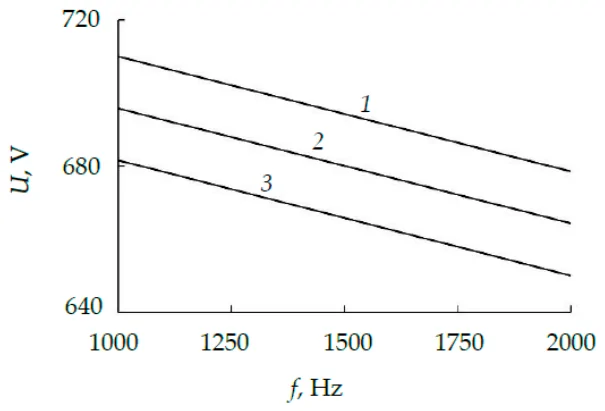
Dependences of the discharge voltage on the pulse repetition rate at *p* = 0.7 Pa, *I* = 1.2 A and τ (in μs): *1*–80; *2*—90; *3*—100.

**Figure 12:**
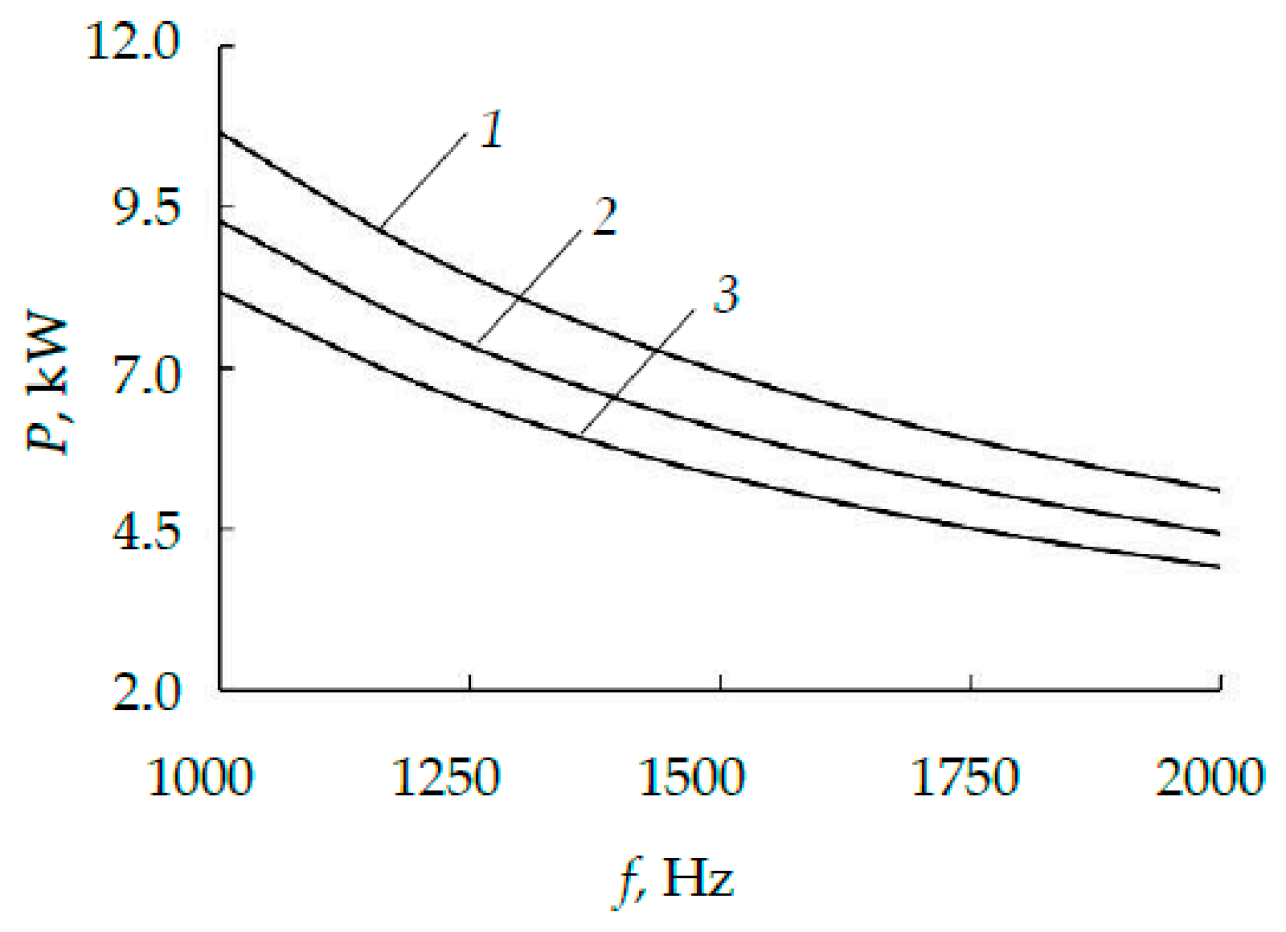
Pulse power dependences on pulse repetition rate at *p* = 0.7 Pa, *I* = 1.2 A and τ (in μs): *1*–80; *2*—90; *3*—100.

The adequacy of model (14), demonstrated using Figure 6, Figure 7, Figure 8, Figure 9 and Figure 10, allows it to be confidently used to predict the influence of other factors on the dependent variables. Figure 11 and Figure 12 show the dependences of discharge voltage $U\left(f\right)=f{\left(f\right)}_{p,\;I,\;\tau =\mathrm{const}}$ and pulse power $P=U\left(f\right)I/\tau f=f{\left(f\right)}_{p,\;I,\;\tau =\mathrm{const}}$, respectively.

**Figure 13:**
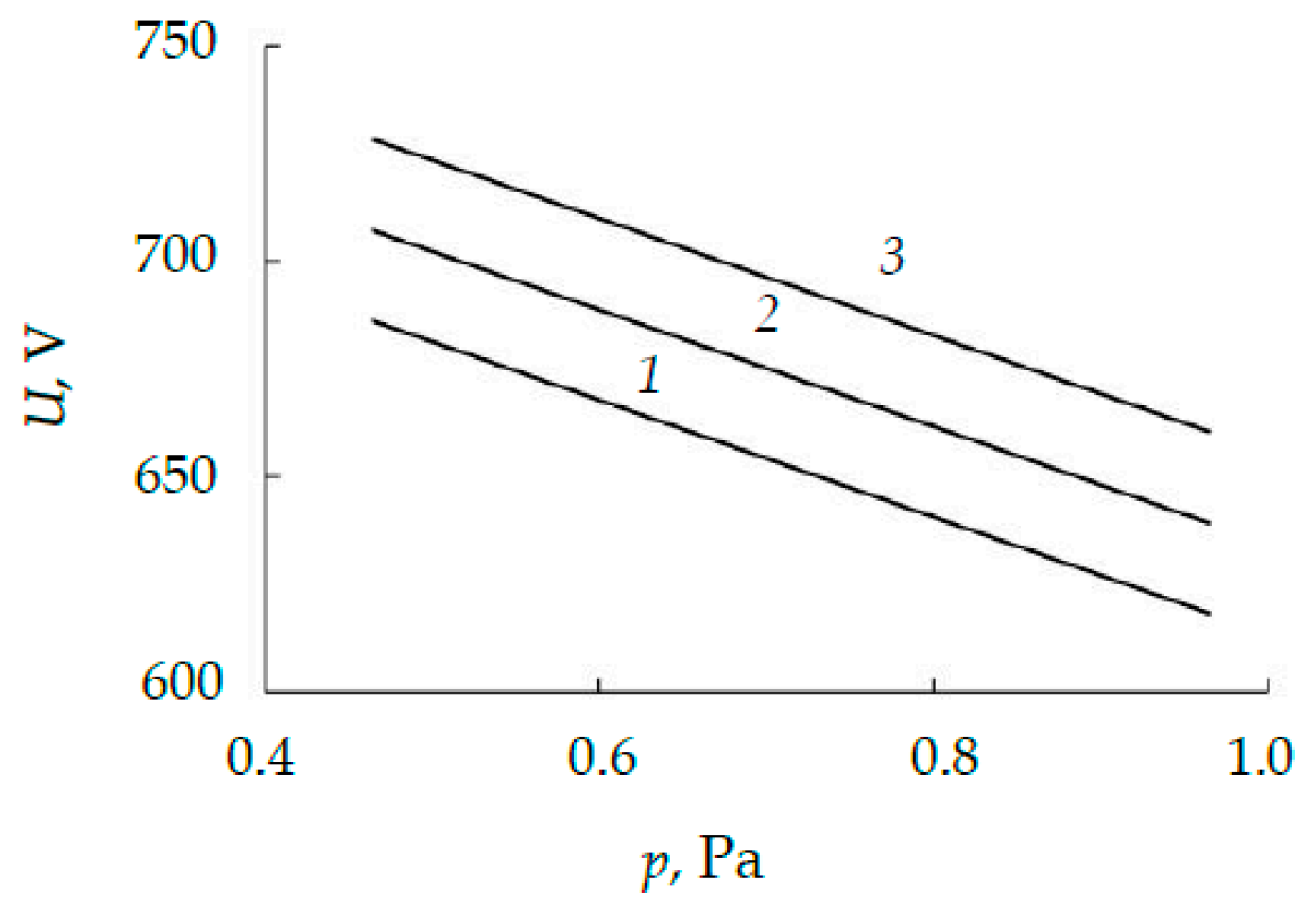
Dependences of discharge voltage on pressure at τ = 90 μs, *f* = 1500 Hz and *I* (in A) *1*–0.8; *2*—1.2; *3*—1.6.

**Figure 14:**
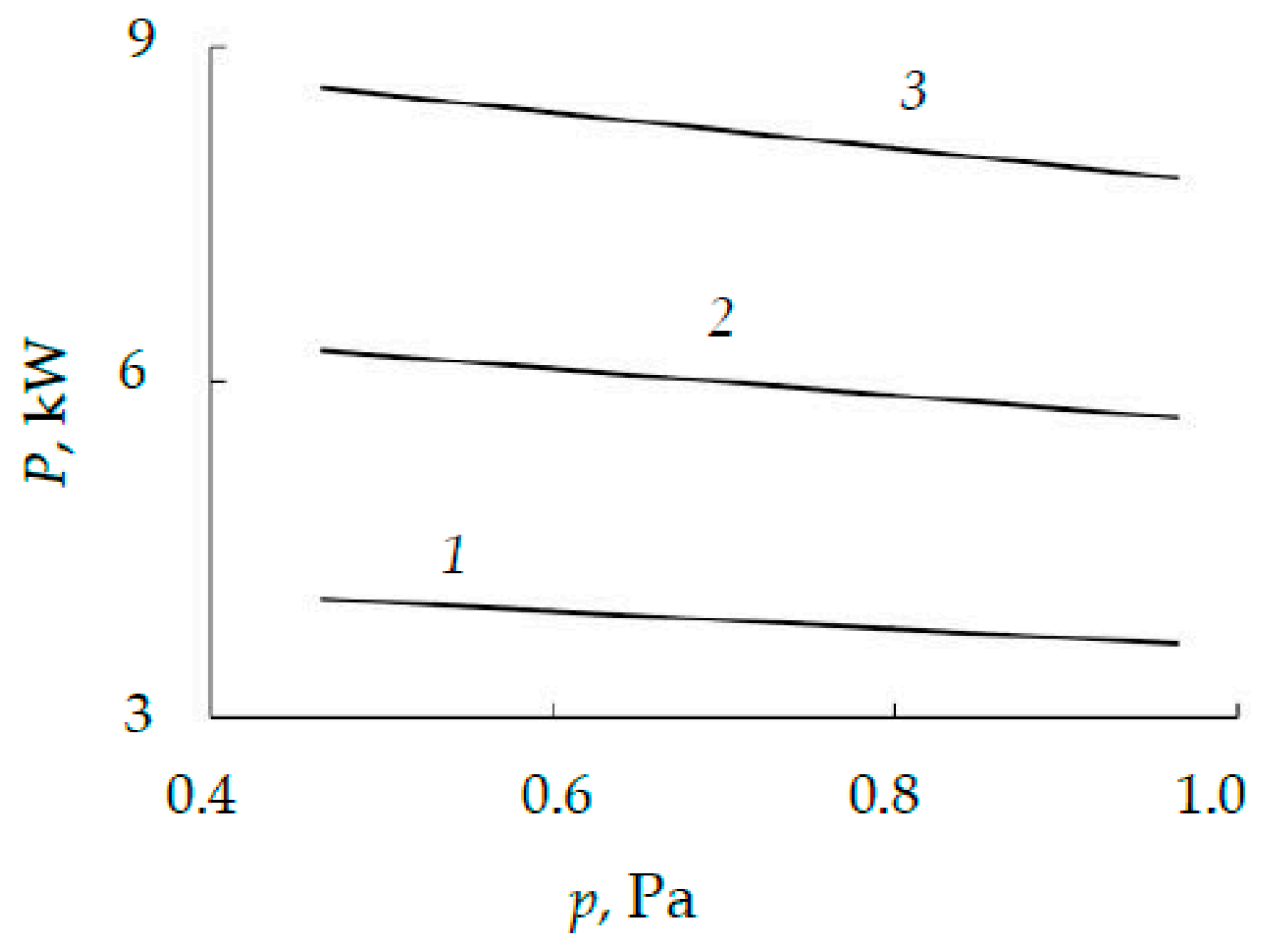
Pulse power dependences on pressure at τ = 90 μs, *f* = 1500 Hz and *I* (in A) *1*–0.8; *2*—1.2; *3*—1.6.

Figure 11 shows that increasing the pulse duration reduces the discharge voltage and, consequently, the pulse energy (see Figure 12). Pulse repetition frequency (see Figure 11 and Figure 12) and operating pressure (see Figure 13 and Figure 14) have similar effects.

## 5. Additional Analysis

**Figure 15:**
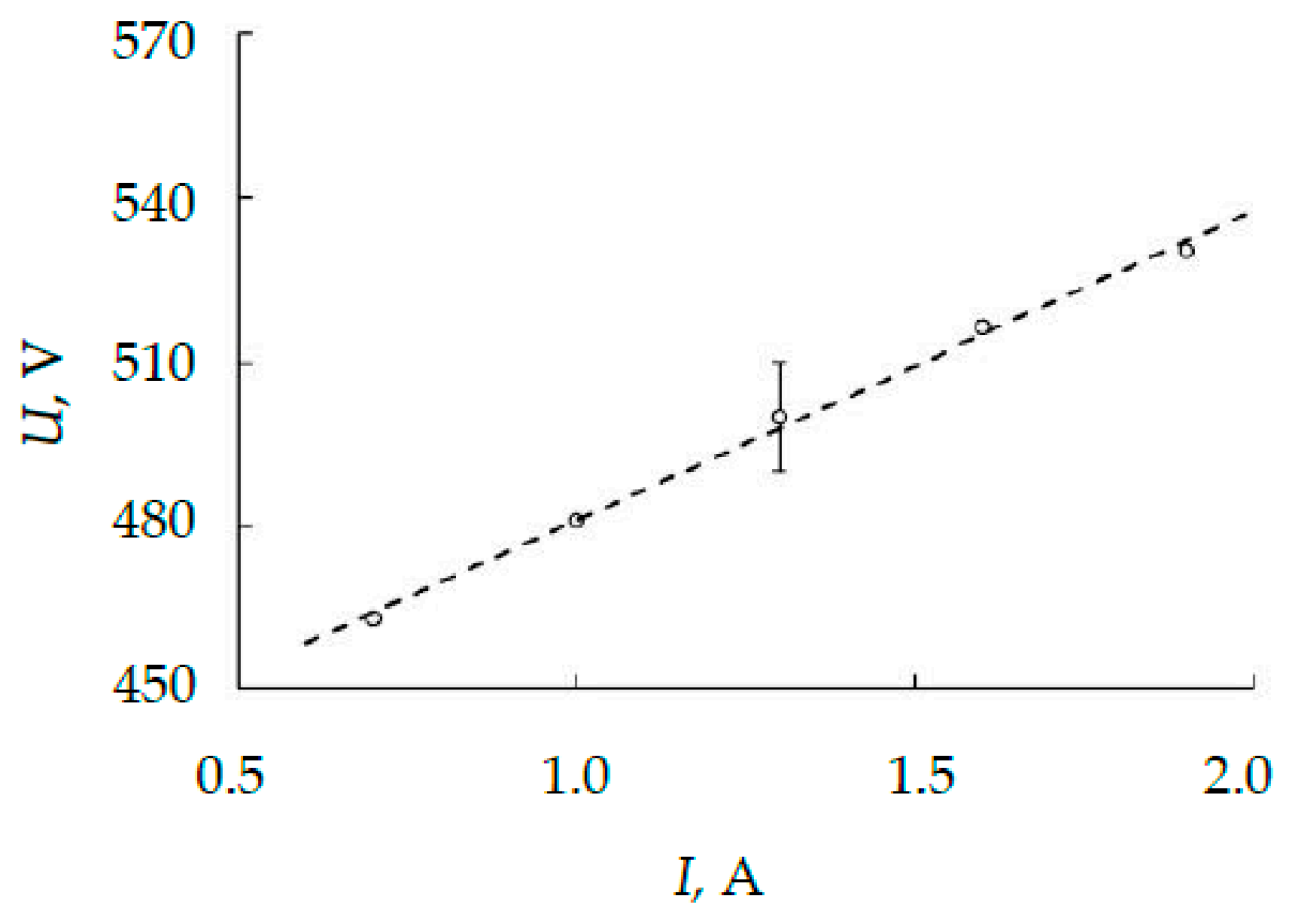
I–V characteristics of a magnetron discharge with a titanium target at *p* = 0.7 Pa, τ = 90 μs, *f* = 1500 Hz; (dots—experiment, dashed line—trend).

We demonstrate that the active-experiment methodology is broadly applicable. It was applied in our study of high-power pulsed magnetron sputtering of a titanium target. One result from the preliminary experiments in this study is shown in Figure 15. The figure includes a first-order polynomial trend line that fits the measurement results with a confidence of at least 0.99. Similar functional relationships occur for the discharge voltage with respect to the other factors considered in this work. For pulse duration this holds for values above 80 μs, and for frequency for values above 1000 Hz. These results justify adopting expression (10) as the mathematical model *U* = *F*(*p*, *I*, τ, *f*). This implies that, for the present problem, a 2^4−1^ fractional-factorial design of the type listed in Table 2 can also be used.

Carrying out the experiment according to the design in Table 2 and using expressions (6) to calculate the quantities $b_{j},\;j=0,\;1,\;\ldots ,\;4$ made it possible to establish that model (10) for a titanium target has the form

*U* = 502 − 17.2*x*_1_ + 18.4*x*_2_ − 15.6*x*_3_ − 18.7*x*_4_. (17)

To test the adequacy of model (17), the random error variance was obtained by analogy with the previous case. Calculations based on ten experiments performed in the center of the plan yielded a value of $\sigma_{U}^{2}\approx 125$. To calculate the adequacy variance, Table 4 was filled in by analogy with Table 3. The sought value is recorded in its last row, $\sigma_{\mathrm{ad}}^{2}\approx 135$. We find the critical value $F_{\alpha ,\;f_{ad},\;f_{U}}$ for α = 0.05, *f*_ad_ = 3, and *f_U_* = 9. It is equal to 3.9 [47]. This value is greater than the ratio $F=\sigma_{\mathrm{ad}}^{2}/\sigma_{U}^{2}=135/125\approx 1.1$, meaning model (17) can be considered adequate. By analogy with the result for a copper target, this conclusion is valid under the assumption that random error is constant in the experimental area.

Comparing models (14) and (17), one can see that the influence of the identified factors on the discharge voltage, determined by the signs of the coefficients $b_{j},\;j=0,\;1,\;\ldots ,\;4$ in both problems, is identical. However, quantitatively, it is, of course, different, due to the different physical properties of titanium and copper.

As for titanium, model (17) can be used to interpolate the discharge voltage within the experimental region, which is limited by the factor values specified in Table 1.

**Figure 16:**
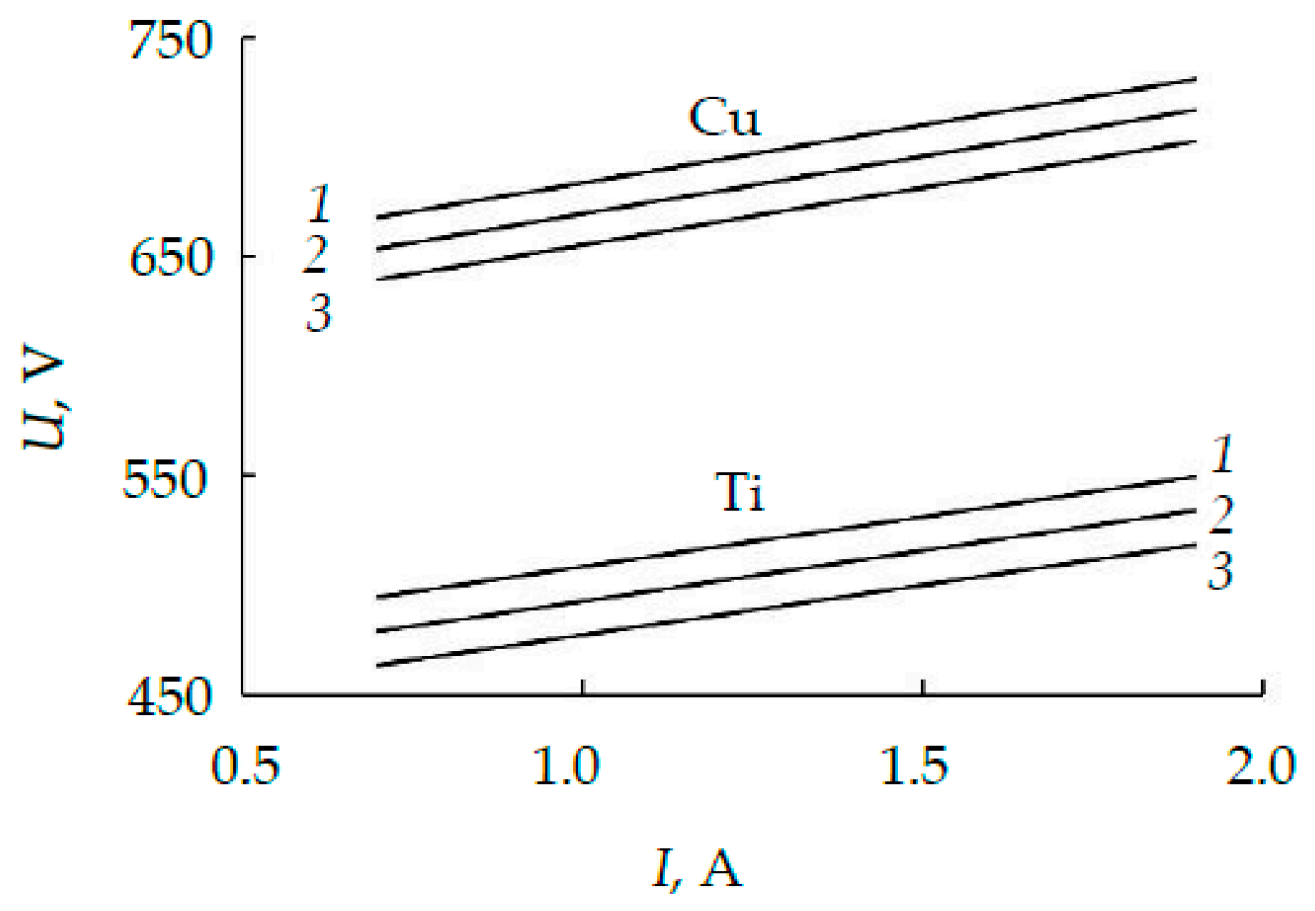
I–V characteristics of magnetron discharge with titanium and copper targets at *p* = 0.7 Pa, *f* = 1500 Hz and τ (in μs): *1*–80; *2*—90; *3*—100.

Next, we compare the influence of factors on the sputtering process of titanium and copper targets, as predicted by models (14) and (17) and obtained using an active experiment. Figure 16 illustrates the I–V characteristics of magnetrons equipped with different targets. The results indicate a discharge voltage of approximately 40% higher for a magnetron with a copper target. This is due to a difference of approximately 40% in the values of the ion–electron emission coefficients of metals. For copper, it is 0.082, and for titanium, 0.113 [48].

**Figure 17:**
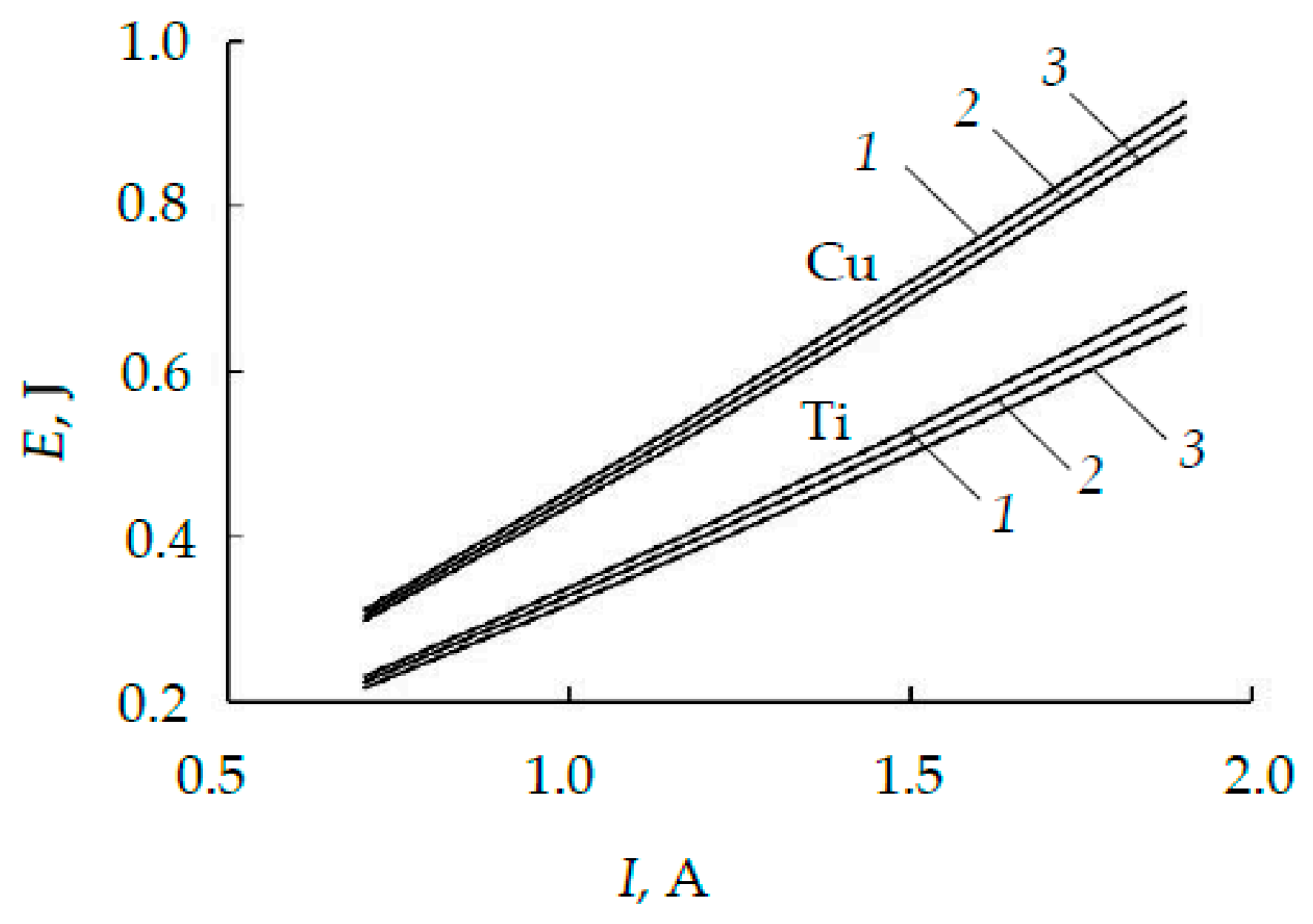
Pulse energy as a function of discharge current during sputtering of titanium and copper targets at *p* = 0.7 Pa, *f* = 1500 Hz and τ (in μs): *1*–80; *2*—90; *3*—100.

**Figure 18:**
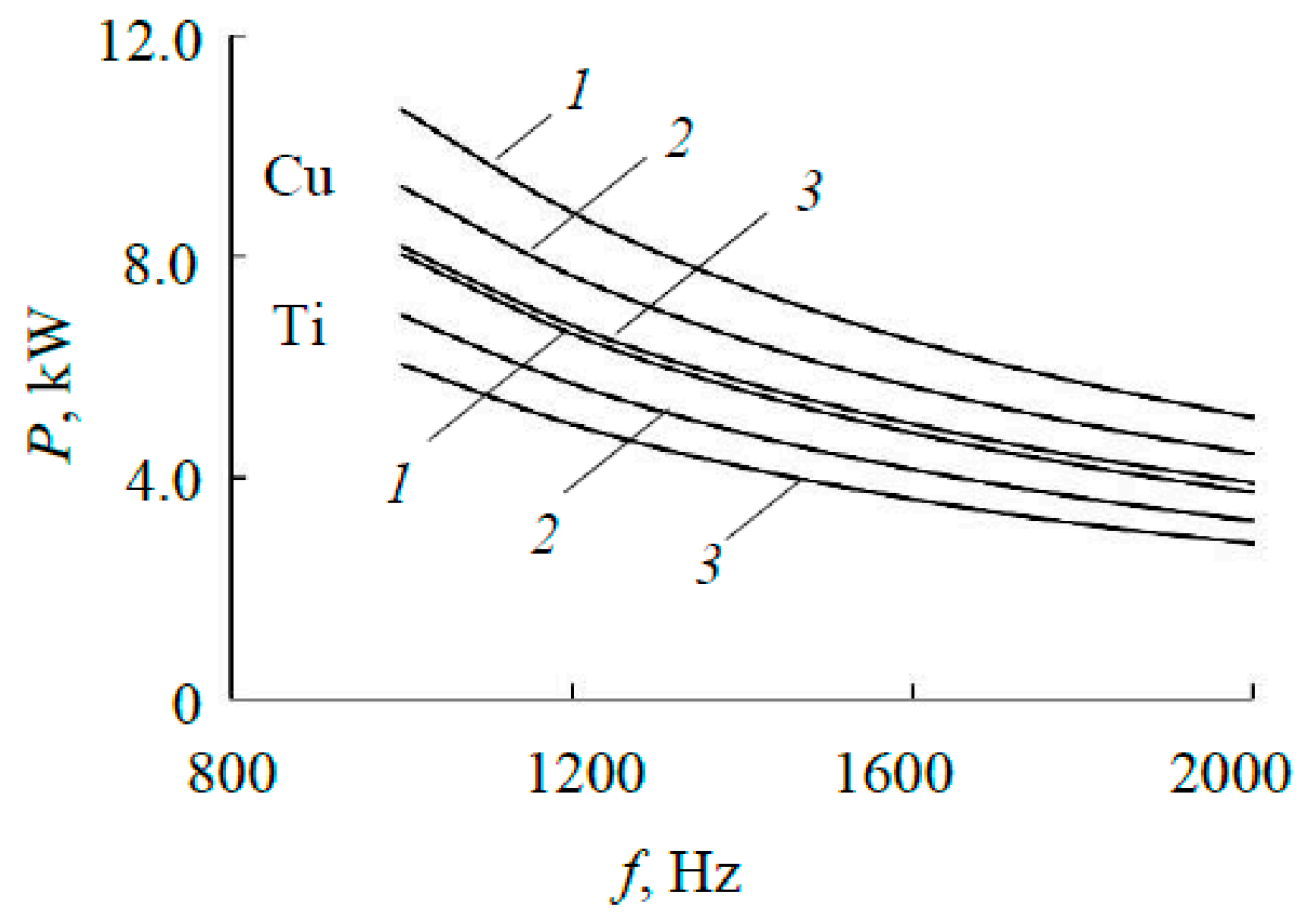
Pulse power dependences on the pulse repetition rate during sputtering of titanium and copper targets, *p* = 0.7 Pa, *I* = 1.2 A and τ (in μs): *1*–80; *2*—90; *3*—100.

Figure 16 and Figure 17 sufficiently reflect the difference between the discharge properties of magnetrons equipped with different targets. In addition, Figure 18 shows a series of dependences of discharge power on the pulse repetition rate. In Figure 18, the relationship between the dependences for titanium and copper targets is preserved; i.e., the power released on a copper target is approximately 40% higher than that on a titanium target. Note that the first-order polynomial models (14) and (17) for the discharge voltage lead to a nonlinear dependence of the discharge power per pulse on the pulse frequency.

**Figure 19:**
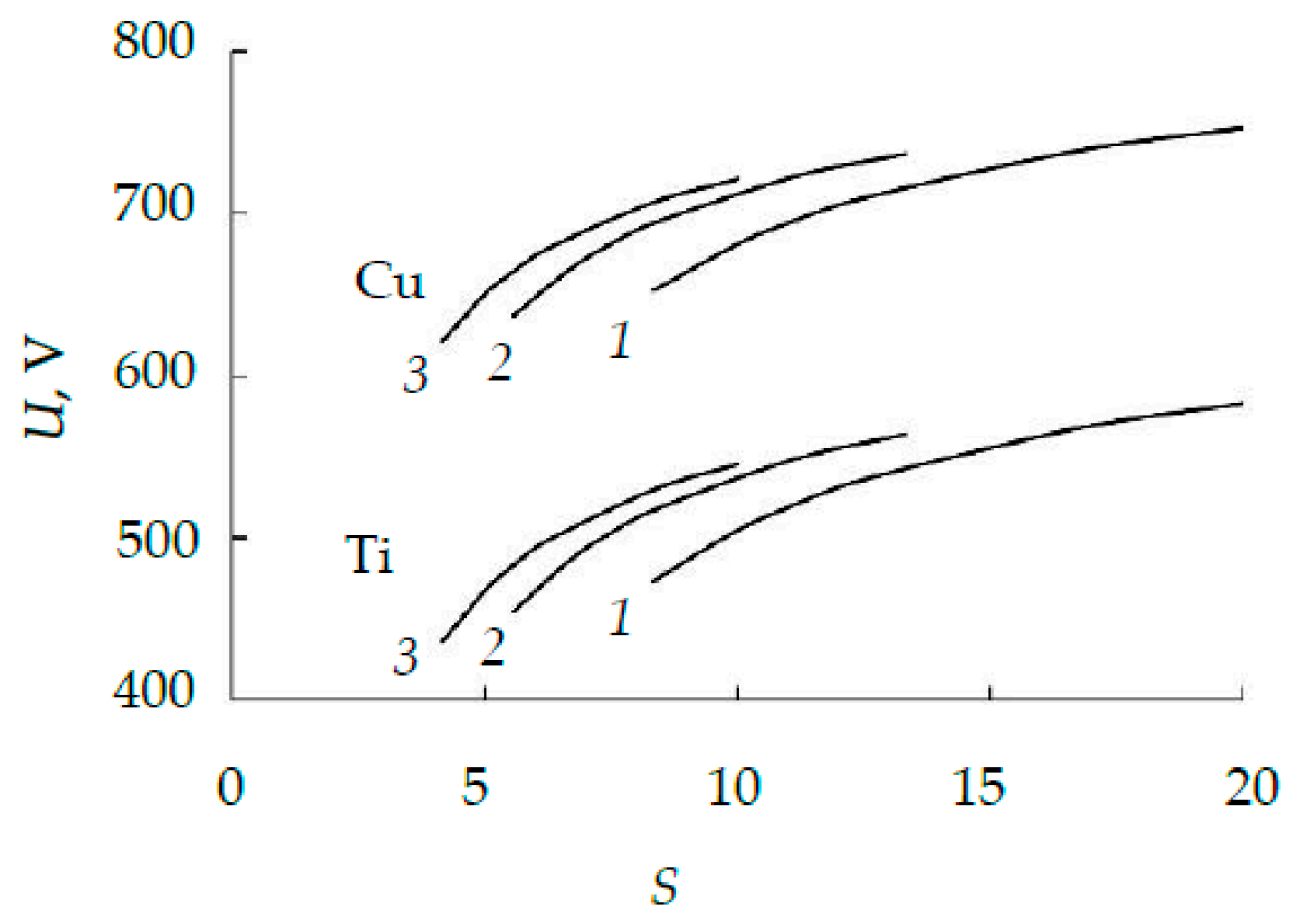
Dependences of the discharge voltage on the pulse fill factor during sputtering of titanium and copper targets, *p* = 0.7 Pa, *I* = 1.2 A and *f* (in Hz): *1*–1000; *2*—1500; *3*—2000.

Of interest is the influence of the pulse fill factor *S* = 1/τ*f* on the sputtering process. As an example, a series of such dependences is shown in Figure 19, which reflects the influence of the fill factor on the discharge voltage. One of these features in the graph in Figure 19 is due to the previously noted difference in the ion–electron emission coefficients of copper and titanium. This difference is shown in Figure 19 as two groups of dependences. Another feature is their significant nonlinearity. Furthermore, not only the pulse fill factor but also their frequency significantly affects the discharge voltage.

**Figure 20:**
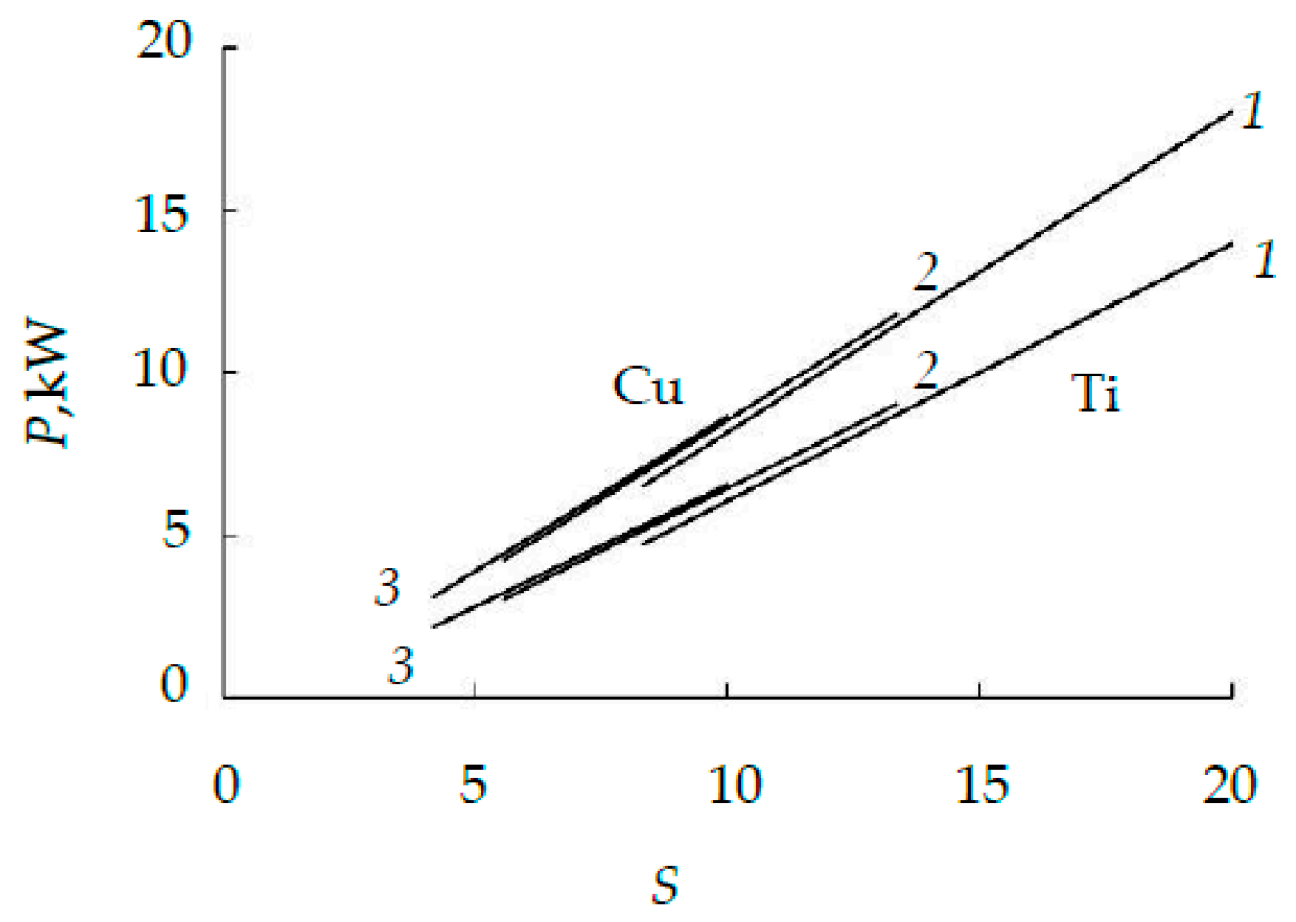
Pulse power dependences on pulse fill factor during sputtering of titanium and copper targets, *p* = 0.7 Pa, *I* = 1.2 A and *f* (in Hz): *1*–1000; *2*—1500; *3*—2000.

However, the pulse power, as follows from Figure 20, is proportional to the fill factor but depends weakly on the pulse repetition rate.

The reader may wonder about the practical value of mathematical model (7). There are at least two types of problems in which this model can be used.

In the first, the dependence of the film growth rate on independent variables may be of interest [31,32]. It is known that it is proportional to the target sputtering yield, which, in turn, is approximately proportional to the ion energy of the sputtering gas. The latter quantity is uniquely related to the discharge voltage.

The other type includes thermal problems in magnetron sputtering, which are described by the Fourier equation [49]. In the steady-state case, one of the boundary conditions is based on the power density on the target surface. Figure 7, Figure 10, Figure 12, Figure 14, Figure 18 and Figure 20 demonstrate the possibility of predicting the values of this quantity in the experimental region.

## 6. Discussion

The objective of this work was an experimental investigation of high-power pulsed magnetron sputtering using an experimental design method. This approach is referred to as an active experiment. It was employed here primarily to study sputtering of a copper target in an argon environment. The discharge voltage was chosen as the primary dependent variable. Independent variables were argon pressure, discharge current, pulse duration, and pulse repetition frequency. Experiments were performed on a balanced cylindrical magnetron. It was found that, within a relatively narrow range of factor variation, the mathematical model relating the variables is well described by a first-order polynomial. Its coefficients were determined from the experimental results obtained using a fractional-factorial design of type 2^4−1^, comprising eight distinct runs. Based on statistical analysis, assuming the persistence of random error in the experimental domain, the adequacy of the model is proved. The model unambiguously quantified the influence of each factor on the discharge: increases in argon pressure, pulse duration, and repetition frequency produced proportional decreases in discharge voltage, pulse power, and pulse energy, whereas an increase in discharge current produced the opposite effect. For comparison, the paper also presents results of analogous experiments performed with a magnetron equipped with a titanium target. The discharge voltage for the copper target was found to be approximately 40% higher than for the titanium target. In the study of the mathematical models, fill factor (duty cycle) was introduced as an additional factor; an increase in fill factor led to an increase in discharge voltage. The results obtained illustrate the effectiveness of the active-experiment technique for studying a particular HiPIMS problem. This allows us to recommend it for studying other tasks that arise when using the HiPIMS method for film deposition. This includes, for example, the study of the dependence of the film growth rate on the selected or more factors.

## Figures and Tables

**Table 1:** Dimensional factors.

Level	*p*, Pa	*I*, A	τ, µs	*f*, Hz
*X_j_*_min_	0.54	0.8	80	1000
*X_j_*_max_	0.80	1.6	100	2000
*X_j_*_0_	0.67	1.2	90	1500
Δ*_j_*	0.13	0.4	10	500

Note: *j* is the factor number; X_j0_ is the value of the *j*-th factor in the middle of the interval (*X_j_*_max_ − *X_j_*_min_); Δ*_j_* is half the width of the interval (*X_j_*_max_ − *X_j_*_min_) of the *j*-th factor.

**Table 2:** Design matrix with measured responses.

Experiment Number	*x*_0_	*x*_1_	*x*_2_	*x*_3_	*x*_4_	*U_i_*, V
1	1	–1	–1	–1	1	676
2	1	–1	–1	1	–1	682
3	1	–1	1	–1	1	717
4	1	–1	1	1	–1	717
5	1	1	–1	–1	–1	668
6	1	1	–1	1	1	610
7	1	1	1	–1	–1	716
8	1	1	1	1	1	654

**Table 3:** Calculation of the adequacy variance.

Experiment Number	*x*_0_	*x*_1_	*x*_2_	*x*_3_	*x*_4_	U_i_*, V	*U_i_,* V	(U_i_ − U_i_*)^2^*,* V^2^
1	1	–1	–1	–1	1	676	676	0
2	1	–1	–1	1	–1	682	679	9
3	1	–1	1	–1	1	717	718	1
4	1	–1	1	1	–1	717	721	16
5	1	1	–1	–1	–1	668	671	9
6	1	1	–1	1	1	610	611	1
7	1	1	1	–1	–1	716	713	9
8	1	1	1	1	1	654	653	1
$\sigma_{\mathrm{ad}}^{2}$, V^2^ (with the number of degrees of freedom *f*_ad_ = 3)								15

**Table 4:** Calculation of the variance of adequacy for the model of the titanium target sputtering process.

Experiment Number	*x*_0_	*x*_1_	*x*_2_	*x*_3_	*x*_4_	U_i_*, V	*U_i_,* V	(U_i_ − U_i_*)^2^, V^2^
1	1	−1	−1	−1	1	495	498	9
2	1	−1	−1	1	−1	516	504	144
3	1	−1	1	−1	1	537	534	9
4	1	−1	1	1	−1	528	540	144
5	1	1	−1	−1	−1	495	500	25
6	1	1	−1	1	1	428	432	16
7	1	1	1	−1	−1	543	537	36
8	1	1	1	1	1	473	469	16
$\sigma_{\mathrm{ad}}^{2}$, V^2^ (with the number of degrees of freedom *f*_ad_ = 3)								135

## Data Availability

The original contributions presented in this study are included in the article. Further inquiries can be directed to the corresponding author.
